# A population representation of the confidence in a decision in the parietal cortex

**DOI:** 10.1016/j.celrep.2025.115526

**Published:** 2025-04-11

**Authors:** Ariel Zylberberg, Michael N. Shadlen

**Affiliations:** 1 Mortimer B. Zuckerman Mind Brain Behavior Institute, Columbia University, New York, NY, USA; 2 Virtual Confidence and Metacognition Laboratory, New York, NY, USA; 3 Department of Neuroscience, Columbia University, New York, NY, USA; 4 The Kavli Institute for Brain Science, Columbia University, New York, NY, USA; 5 Howard Hughes Medical Institute, Chevy Chase, MD, USA; 6 Grossman Center for the Statistics of the Mind, Columbia University, New York, NY, USA; 7 X (formerly Twitter): @azylb; 8 Lead contact

## Abstract

Many decisions arise from a race between competing evidence accumulation processes that terminate upon reaching a threshold. We ask whether neurons supporting this accumulation also encode confidence—whether a choice is correct or incorrect. Monkeys performed a reaction-time random dot motion task while populations of neurons were recorded from the lateral intraparietal (LIP) area. Shortly before the choice report, LIP neurons with response fields overlapping the contralateral choice target ($T_{\text{in}}$ neurons) convey information about choice accuracy. We show that this information would give rise to the behavioral signatures of confidence observed in humans. These findings are surprising because the activity of $T_{\text{in}}$ neurons just before the report is, on average, independent of reaction time and motion strength—strong predictors of accuracy. This tension is resolved by considering the heterogeneity of neuronal responses across the population of $T_{\text{in}}$ neurons. We conclude that neurons representing evidence accumulation may inform a decision-maker’s confidence.

## In brief

At the end of a decision based on evidence accumulation, the neural representation of the accumulation is invariant across decisions. Zylberberg and Shadlen show, nonetheless, that accuracy can be decoded from the heterogeneous dynamics of the contributing neurons. The predicted accuracy replicates behavioral signatures of confidence in humans.

## Graphical abstract

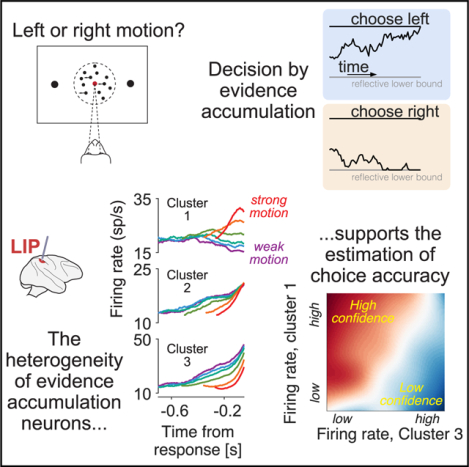

## INTRODUCTION

Choice, reaction time (RT), and confidence are often considered the pillars of choice behavior; a comprehensive model should account for all three. However, the most prevalent models of binary decision-making, signal detection theory (SDT) and the drift-diffusion model (DDM), account for only two. In SDT, the sign of the difference between a sample of evidence and a decision criterion determines choice; confidence increases with that difference’s magnitude.^1,2^ Because SDT frames the decision as single-sample categorization, it cannot account for RTs except by positing that evidence closer to the criterion leads to slower choices.^3^ In the DDM, the decision is made by accumulating samples of evidence over time.^4–7^ The decision ends when the accumulated evidence exceeds an upper or lower bound. The model naturally accounts for choice and RT but lacks a straight-forward explanation of confidence. This is because accumulated evidence at choice time is uninformative about accuracy.

In the brain, simple binary decisions are implemented as a race between two competing evidence-accumulation processes.^8,9^ The first process to reach an upper bound determines choice and RT. A case in point is the random dot motion task, in which monkeys make binary decisions about motion direction and communicate their decision with a saccadic eye movement. Neurons in the lateral intraparietal (LIP) area with response fields overlapping the choice-target contralateral to the recording site ($T_{\text{in}}$ neurons) represent the accumulation of evidence in favor of contralateral target selection.^10,11^ Similar neural responses exist in other brain areas and species.^9,12–16^ The decision process is well captured by race models, in which each choice alternative is represented by a separate evidence-accumulation process.^17,18^ In trials where the monkey chooses the contralateral target (i.e., the one within the neurons’ response field), the $T_{\text{in}}$ neurons represent the winning race, whereas on ipsilateral choices, they represent the losing race.

Race models offer the leading explanation of choice, RT, and confidence in simple binary decisions. Confidence is thought to be guided by the difference between the winning and the losing race–what is known as the “balance of evidence” hypothesis.^19^ Since, at the moment of choice, the winning race is at its upper bound, confidence is determined by how far the losing race is from its bound. Models embracing the balance of evidence hypothesis account for how confidence covaries with evidence strength, RT, and accuracy.^19–31^

Here, we explore the alternative hypothesis that confidence depends not on the difference between drift-diffusion processes but on systematic variations within the neural populations that collectively manifest these processes. Each competing drift-diffusion process presumably arises from the average activity over a large population of neurons with similar response properties.^11,32^ Theoretical studies suggest that confidence may be informed by the variability in firing rates among neurons supporting the winning race, but this hypothesis remains untested.^33,34^

We reanalyze data from large population recordings in the macaque LIP in a decision-making task.^11,35^ We tested a key prediction of balance of evidence models, namely that the losing race at decision termination contains more information about accuracy than the winning race. We use the $T_{\text{in}}$ neurons to predict choice accuracy in a 100 ms window preceding the saccade. Contrary to the prediction of balance of evidence models, choice accuracy information is more strongly encoded by neurons representing the winning race than by those representing the losing race. These accuracy predictions also exhibit signatures of confidence reports observed in human behavioral studies.

These findings are unexpected because the average firing rate of $T_{\text{in}}$ neurons reaches a stereotyped level just before a saccadic eye movement to the contralateral choice target. However, the average firing rate belies considerable heterogeneity across the population. Not all $T_{\text{in}}$ neurons reach a common activity level. Instead, some encode evidence strength, while others track elapsed decision time. Through clustering analysis, we demonstrate that choice accuracy information is distributed across these two $T_{\text{in}}$ sub-populations. Thus, the heterogeneous activity of $T_{\text{in}}$ neurons enables a linear readout of the confidence that the choice is correct.

## RESULTS

### Task, behavior, and neurophysiological recordings

**Figure 1.**
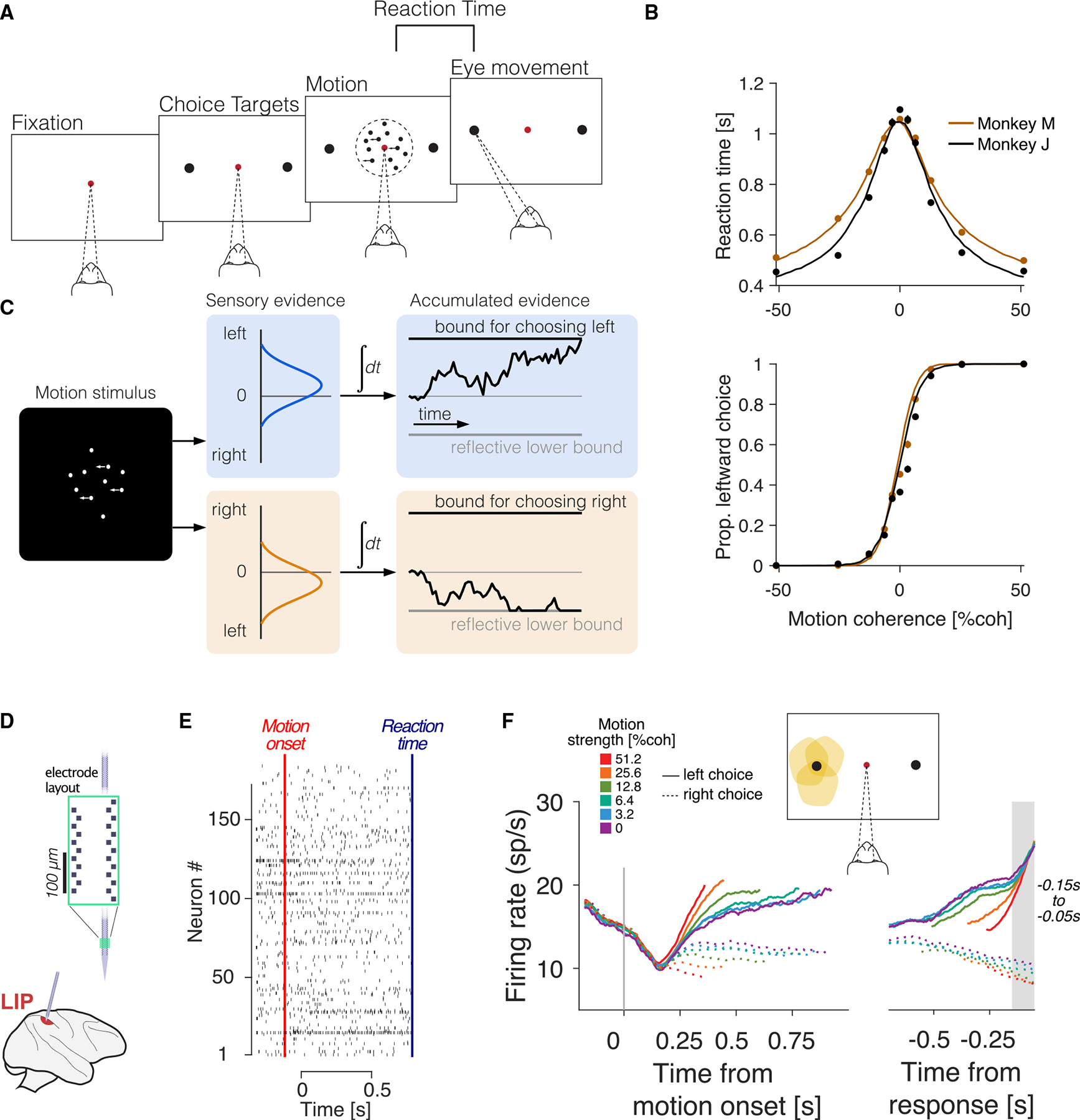
Large-scale recordings from LIP in a decision-making task (A) Sequence of events in the random dot motion task. The monkey fixates on a central spot, and then two choice targets appear, followed by the random dot motion stimulus after a random delay. The monkey reports its decision via a saccadic eye movement to one of the targets and is rewarded for correct choices (left or right target for leftward or rightward motion, respectively). In noise-only (0% coherence) trials, the reward is given with probability $\frac{1}{2}$. (B) Psychometric functions for the two monkeys studied by Steinemann et al.^11^ The average RT (top) and proportion of leftward choices (bottom) are plotted against motion coherence. Positive and negative coherence indicate leftward and rightward motion, respectively. Solid lines are fits of the race model shown in (C). Error bars: SE. (C) Sketch of the race model. The random dot motion stimulus provides sequential samples of momentary evidence for right minus left and left minus right, accumulated over time in two drift-diffusion processes. Samples are drawn from anti-correlated normal distributions with means of opposite sign and proportional to motion strength. The decision terminates when one process reaches its bound, determining choice and decision time. A lower reflective bound constrains negative accumulation. RT is the sum of decision time and a normally distributed non-decision time. (D) Schematic of the Neuropixels probe used to record LIP activity in the right hemisphere (both monkeys). (E) Raster plot of 191 simultaneously recorded neurons in a representative trial. Red and blue vertical lines mark motion onset and saccade initiation, respectively. (F) Average response of $T_{\text{in}}$ neurons (N=152) aligned to motion onset (left) and saccade initiation (right). Motion strength is color coded (legend). Solid and dashed lines represent leftward and rightward motion, respectively (correct trials only). Gray shading marks 150–50 ms before saccade initiation, the focus period for most analyses. Reproduced from Steinemann et al.^11^

We analyzed previously published Neuropixels recordings from the LIP of two rhesus monkeys (*Macaca mulatta*) performing the random dot motion task.^11,35^ The monkeys indicated their choices by redirecting their gaze from a central fixation point to a left or right choice target. Monkeys were allowed to indicate their decision when ready, thus giving rise to two behavioral measures: choice and RT (Figure 1A). The degree of difficulty was controlled by the motion coherence, defined as the probability that a dot displayed at time t will be redrawn in the direction of motion when replotted 40 ms later as opposed to being randomly repositioned. On each trial, motion coherence was selected pseudorandomly from the list {±0%, ±3.2%, ±6.4%, ±12.8%, ±25.6%, and ±51.2%}. The sign of the motion coherence indicates the direction (leftward for positive values). For the 0% coherence motion, the sign denotes the random direction to be rewarded on that trial. The proportion of leftward choices increases with motion coherence, and RT shortens as a function of motion strength (Figure 1B). In about half of the trials, a brief (100 ms) pulse of motion, equivalent to a small change in motion coherence, was presented at a random time.

The relationship between choice, RT, and motion coherence is consistent with the race model described above and depicted in Figure 1C. The RT is the sum of the decision time and a non-decision time. In our instantiation of the race model, the drift-diffusion processes cannot fall below a lower reflective bound, enforcing non-negative firing rates.^36^

In addition to the main task, the monkeys made visually guided and memory-guided saccades to peripheral targets after variable delays (see STAR Methods).^37–39^ The task served to identify, post hoc, neurons with persistent activity during planning of contralateral saccades ($T_{\text{in}}$ neurons). Monkeys also performed a passive motion viewing task in which they were rewarded for maintaining fixation while viewing random dot motion (higher motion strengths only; see STAR Methods).

The neural data^11,35^ were recorded from the LIP using high-density non-human primate (NHP)-Neuropixels probes. Between 54 and 203 single neurons were recorded simultaneously over eight sessions (Figure 1E). Steinemann et al.^11^ showed that $T_{\text{in}}$ neurons represent the drift-diffusion signal associated with stochastic choice and RT in single trials. On average, $T_{\text{in}}$ neurons ramp with positive slope in trials where the monkey chooses the target in the neurons’ response field, and this slope is steeper as a function of motion strength (Figure 1F).^10^ Importantly, the population of $T_{\text{in}}$ neurons reaches a common level of activity ~100 ms before a saccadic eye movement toward the contralateral choice target. These observations conform to the predictions of the race model illustrated in Figure 1C, under the assumption that the $T_{\text{in}}$ neurons represent the accumulation of evidence for a contralateral choice and that another (unobserved) population of neurons represents the accumulation of evidence for the ipsilateral (rightward) choice.^17,40^

### Choice accuracy decoded from LIP population activity

We investigated whether $T_{\text{in}}$ neurons, which represent the accumulation of noisy evidence, are also predictive of whether the choice would be correct, potentially informing confidence or reward prediction. We trained a logistic decoder to predict the accuracy of each contraversive (i.e., left) choice from the activity of the $T_{\text{in}}$ neurons in a presaccadic window (150–50 ms window before choice report):

(Equation 1) logitpcorrect=βconfS˜⊤Tin+β0,

where $\beta_{\text{conf}}$ is a column vector of regression coefficients (one per $T_{\text{in}}$ neuron) and $\beta_{0}$ is a bias term. ${\tilde{S}}_{\text{Tin}}$ contains the standardized spike count of each $T_{\text{in}}$ neuron in the presaccadic window. The model is fit separately for each session and choice category, including both correct and error trials. The logistic decoder is trained with 10-fold cross-validation: we divide the data into 10 groups using one for prediction and the remaining 9 for training, repeating the process so that confidence estimates for every trial are out of sample.

**Figure 2.**
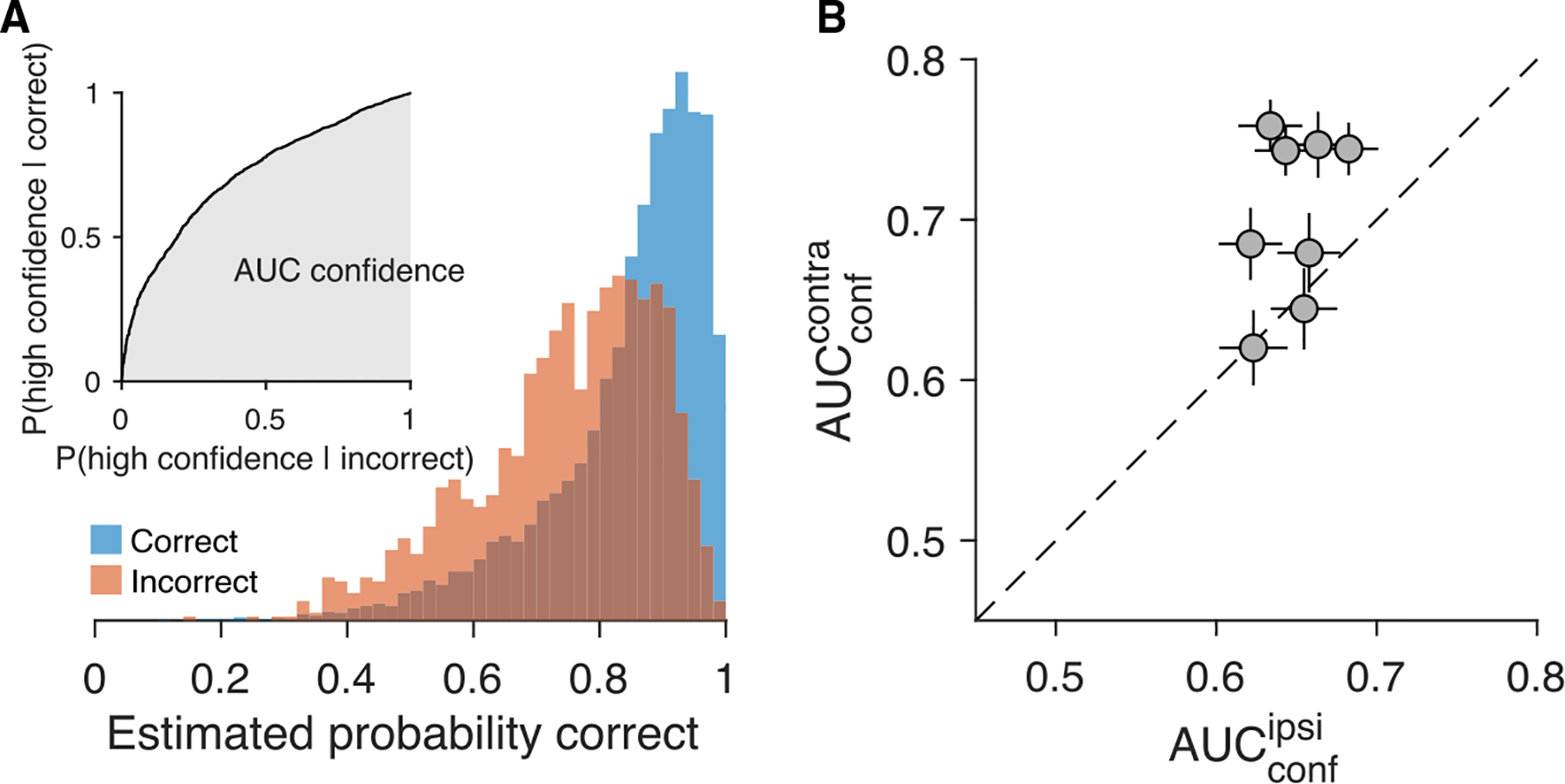
Confidence inferred from the population of $T_{\text{in}}$ neurons (A) Confidence estimates from Equation 1 for correct (blue) and incorrect (red) choices. These distributions generate an ROC curve (inset). The area under the ROC (AUCconf; gray) estimates the probability correct (accuracy). Data are pooled from eight sessions. Only contralateral (left) choices are included. (B) ${\text{AUC}}_{\text{conf}}$ values for trials where the ipsilateral (abscissa) or contralateral (ordinate) target was chosen. Each point represents a session. Error bars: SE (bootstrap).

We use receiver operating characteristic (ROC) analysis to assess how effectively the predicted probability correct (Equation 1) distinguishes correct from incorrect choices (Figure 2A). The area under the ROC curve (${\text{AUC}}_{\text{conf}}$) quantifies the probability that, given one correct and one incorrect choice, the predicted probability correct is greater for the correct one. Using this simple metric, we can evaluate the balance of evidence hypothesis mentioned above.

We compared ${\text{AUC}}_{\text{conf}}$ derived from logistic models fit separately for contralateral and ipsilateral choices. $T_{\text{in}}$ neurons represent the winning race for contralateral and the losing race for ipsilateral choices. Contrary to the balance of evidence hypothesis, $T_{\text{in}}$ neurons encode more information about accuracy for contralateral choices (Figure 2B) (p=0.008, one-tailed t test).

The finding is surprising because $T_{\text{in}}$ neurons reach a stereotyped activity level before contralateral choices, independent of motion strength and RT (Figure 1F).^10^ Since motion strength and RT are strong predictors of accuracy, one would not expect $T_{\text{in}}$ neurons to contain information about choice accuracy in the presaccadic window. We will address this tension after bolstering the claim that choice accuracy inferred from neural activity replicates behavioral features of confidence identified in human experiments.

### Choice accuracy inferred from neural activity reproduces behavioral features of confidence

**Figure 3.**
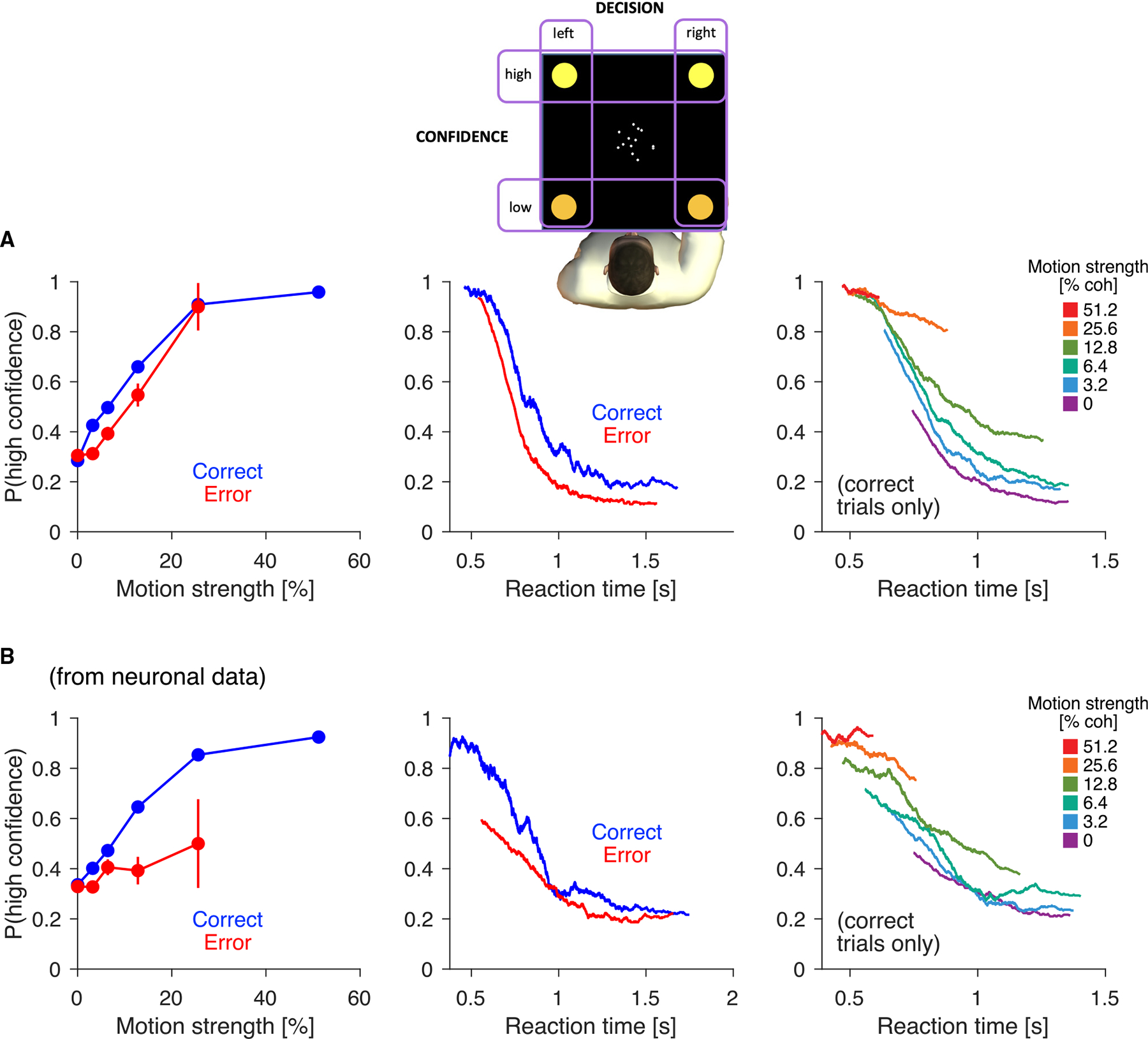
Choice accuracy inferred from neural activity reproduces behavioral signatures of confidence (A) Random dot motion task with simultaneous choice and confidence reports from van Den Berg et al.^22^ In alternating blocks, the top or bottom two targets signaled high confidence, while the remaining two signaled low confidence. Data are from a representative participant (N=9,024 trials; 61% high-confidence choices). Data from the other participants are shown in Figure S1. Left: proportion of high-confidence choices by motion strength, separately for correct and incorrect choices (conditions with fewer than 4 trials were excluded). Reproduced from van Den Berg et al.^22^ Middle: proportion of high-confidence choices by RT, separately for correct and incorrect choices. Trials were sorted by RT and smoothed (boxcar filter, N=300 trials). Right: same as middle but for correct trials only, plotted separately for each motion strength. (B) Same analyses as in (A) but for the confidence inferred from $T_{\text{in}}$ neural activity. The continuous confidence estimate was thresholded to match the behavioral data (A). A version with non-thresholded confidence estimates is included as Figure S2A.

The monkeys did not report their confidence, so we cannot establish a correlation between the putative confidence signal and behavior. Instead, we ask whether a monkey exploiting this signal would mimic the regularities of human confidence reports in a similar task. van Den Berg et al.^22^ asked human participants to perform a variant of the random dot motion task in which they reported choice and confidence (high/low) simultaneously by moving a handle to one of four targets (Figure 3A). The results from a representative participant are shown in Figure 3A, demonstrating that (1) confidence is greater for correct than incorrect decisions (Figure 3A, left); (2) for correct decisions, confidence increases with motion strength (Figure 3A, left); (3) for incorrect decisions, confidence also increases with motion strength (Figure 3A, left); (4) confidence decreases with RT (Figure 3A, middle); (5) for a given RT, confidence is lower for incorrect than correct decisions (Figure 3A, middle); and (6) for a given RT, confidence increases with motion strength (Figure 3 A, right).

The putative confidence signal reproduces these observations. To parallel the design of van Den Berg et al., we thresholded the putative confidence signal (Equation 1) using a criterion set such that the proportion of high-confidence choices matches the human experiment (61% high-confidence choices). Without any free parameters, the confidence signal qualitatively reproduces all the behavioral hallmarks of confidence observed by van Den Berg et al. (Figure 3B). These regularities are also observed without thresholding (Figure S2).

In short, the population activity of $T_{\text{in}}$ neurons measured just before a contralateral choice report contains information bearing on whether the choice is correct or incorrect. The confidence signal varies with motion strength, RT, and accuracy in a similar manner to explicit confidence reports. Importantly, it is not necessary to consider the state of the losing race or the decision time to reproduce the behavioral features of confidence; the necessary information is contained in the population response of the $T_{\text{in}}$ neurons.

Because of the tight link between confidence and RT,^24,31,41^ we reasoned that the population activity of $T_{\text{in}}$ neurons just before the response should contain information about RT. We fit a regression model to the RTs for trials with a contralateral choice, again using the spike counts of the $T_{\text{in}}$ neurons in the presaccadic window (Equation 8). If all $T_{\text{in}}$ neurons reach a stereotyped level of activity before the response, then there should be no information about RT just before the choice. Contrary to this expectation, we were able to reliably distinguish longer from shorter RTs (relative to the median) from the population activity of $T_{\text{in}}$ neurons (AUC = 0.85 ± 0.02; mean ± SE across sessions). Thus, shortly before the choice report, $T_{\text{in}}$ neurons contain information about the time taken to make the decision. This information likely contributes to the ability of the accuracy decoder to reproduce the behavioral features of confidence that are thought to require an explicit representation of decision time.

We conducted a control analysis to assess whether the prediction of choice accuracy from neural activity could be attributed to differences in the amplitude of the eye movement used to report the choice. This scenario seemed unlikely *a priori*, as the epoch ends 50 ms before saccade initiation, when the monkey is still holding fixation. To test this, we repeated the decoding analysis, substituting the neural signals in Equation 1 with the x and y coordinates of the gaze averaged over two intervals: 100 ms before saccade detection and 100 ms after saccade completion (yielding four regressors). The capacity to decode choice accuracy from these signals was significantly lower than that achieved with $T_{\text{in}}$ neurons $\left({\text{AUC}}_{\text{conf}}=0.57\pm 0.01\right.$ vs. 0.72 ± 0.01, respectively; $p<10^{-6}$, bootstrap). Moreover, gaze signals failed to reproduce the behavioral characteristics of confidence (Figure S2B).

### Heterogeneity of $T_{\text{in}}$ responses underpins the representation of choice accuracy

The observation that $T_{\text{in}}$ neurons achieve a stereotyped state at the end of a contralateral choice seems incompatible with a graded representation of choice accuracy. However, this stereotyped state is evident in firing rates averaged over many neurons.^10,11^ We thus considered the possibility that the population of $T_{\text{in}}$ neurons might contain information about choice accuracy that is lost in the firing rate averages.

**Figure 4.**
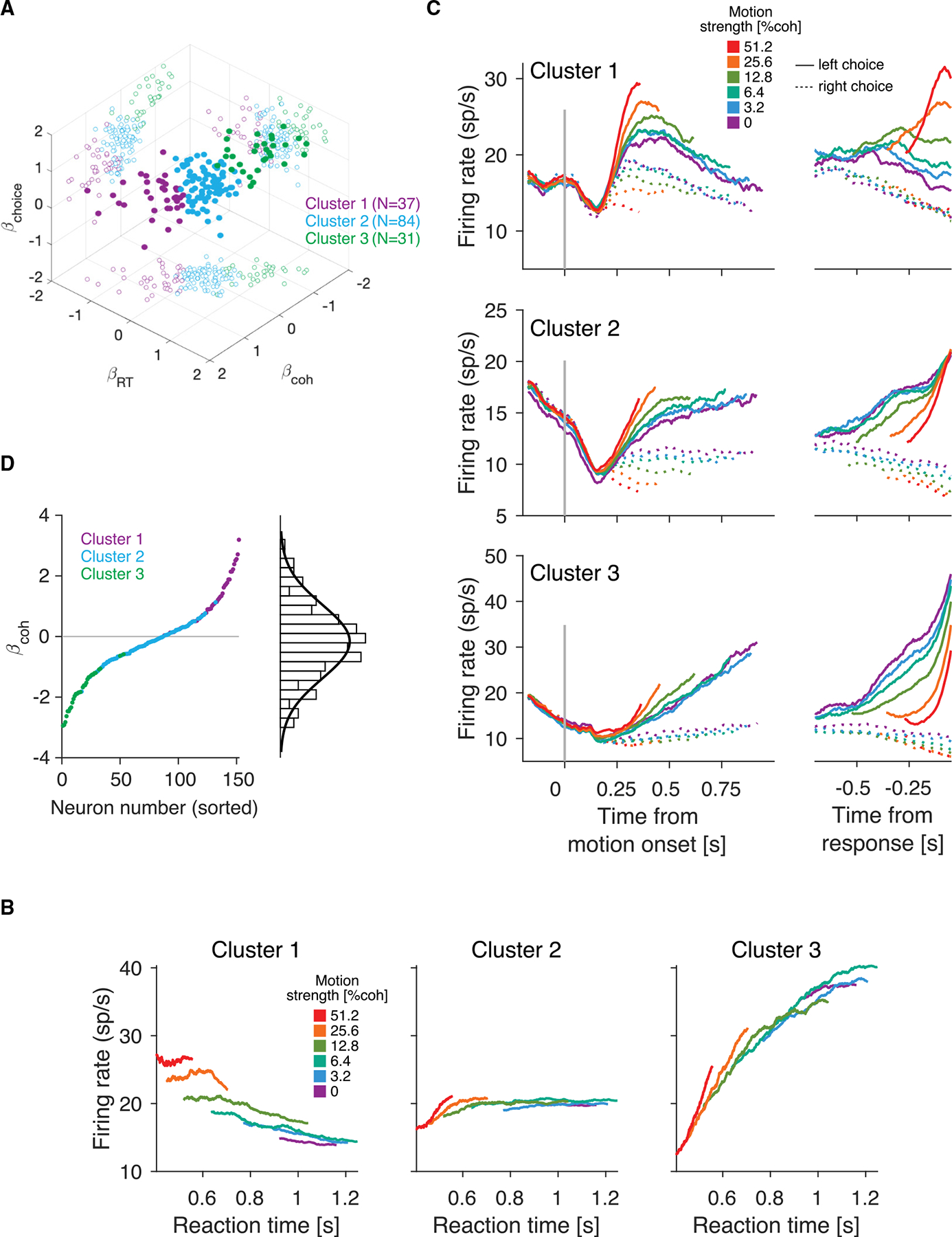
Distinct response characteristics of $T_{\text{in}}$ neurons **$T_{\text{in}}$** neurons differ in their representation of motion coherence, RT, and choice. We identified three clusters using a linear regression model and k-means clustering. (A) Regression coefficients for choice, motion coherence, and RT for each neuron. Each filled symbol represents one neuron, colored by cluster membership. Open circles show two-dimensional (2D) projections. N, number of neurons in the cluster (color). (B) Average firing rate within each cluster as a function of RT, computed within the presaccadic window. Traces are separated by motion strength and include only correct contralateral choices (smoothed with a boxcar filter, N=500 trials). (C) Average response of $T_{\text{in}}$ neurons by cluster. The same conventions are used as in Figure 1F. (D) Regression coefficients for motion coherence from a linear regression predicting spike counts (Z scores) in the presaccadic window. Colors indicate cluster membership. The histogram (right) of regression weights is well described by a normal distribution (black trace) with mean = −0.17 and SD = 1.2.

To test this possibility, we used a combination of linear regression and k-means clustering. The linear regression sets out to explain the spike counts in the presaccadic window for each $T_{\text{in}}$ neuron on each trial using three variables, the motion coherence, choice, and RT, plus an offset (Equation 9). We fit the model independently for each neuron and applied k-means to assign the neurons to three clusters. Clusters were determined using the regression coefficients for these variables (Figure 4A).

The $T_{\text{in}}$ neurons cluster into groups that exhibit distinct response characteristics (Figure 4B). Just before the choice is reported, neurons in cluster $1\left(T_{\text{in}}^{k=1}\right)$ exhibit higher firing rates for strong motion and faster choices. The traces corresponding to different motion strengths do not converge when conditioned on RT, indicating that the activity of these neurons at the moment of choice is informative about both RT and motion strength. In contrast, neurons in cluster $2\left(T_{\text{in}}^{k=2}\right)$ appear to be largely unaffected by RT or motion strength, consistent with expectations for neurons that reach a stereotyped level of activity prior to response. For the neurons in cluster $3\left(T_{\text{in}}^{k=3}\right)$, the activity increases strongly with RT and is only slightly influenced by motion strength. We repeated the clustering analysis using only the oddnumbered trials or only the even-numbered trials from each session. Applying k-means to the coefficients derived from odd and even trials, we observed that most neurons (89%) were assigned to the same cluster in both realizations, indicating that the neuron clustering is robust (Figure S3).

All three clusters exhibit choice selectivity; compare the solid and dashed traces in Figure 4C and responses for correct and error choices (Figure S5A). Neither $T_{\text{in}}^{k=1}$ nor $T_{\text{in}}^{k=3}$ neurons reach a common level of activity prior to the response. $T_{\text{in}}^{k=1}$ neurons do not show the ramping activity usually associated with $T_{\text{in}}$ neurons. Instead, their firing rate traces are parallel across motion coherences, resembling the momentary evidence representation in upstream visual areas.^42^ For contralateral choices, $T_{\text{in}}^{k=3}$ neurons appear to increase their firing rate more rapidly than $T_{\text{in}}^{k=2}$ or $T_{\text{in}}^{k=1}$ neurons. The firing rate of $T_{\text{in}}^{k=3}$ is higher just before choice in difficult (i.e., low motion strength) decisions compared to easier ones.

The latency to choice selectivity was similar across clusters. We calculated this latency independently for each neuron using the cumulative sum (CUSUM) method.^43,44^ The average latency to direction selectivity values were 0.22 ± 0.03, 0.22 ± 0.02, and 0.23 ± 0.03 s from motion onset for neurons in clusters 1, 2, and 3, respectively. The clusters showed no significant latency differences (Figure S4) (p>0.3 for all three pairwise comparisons, t test).

The heterogeneity of neuronal responses across the population of $T_{\text{in}}$ neurons can also be observed by analyzing individual neurons, without clustering. We used a linear regression model to characterize the relationship between motion coherence and the neuronal activity in the presaccadic window. Motion coherence for each trial (plus an offset) was used to explain the standardized (Z scored) spike counts in the presaccadic window. We included only trials with a contralateral choice. The regression coefficient associated with motion coherence varies substantially between neurons (Figure 4D). For some neurons, activity increases with motion coherence ($\beta_{\text{coh}}>0$), while for others, it decreases ($\beta_{\text{coh}}<0$). The values of $\beta_{\text{coh}}$ are approximately normally distributed (Figure 4D). Because the mean is close to zero, the activity of $T_{\text{in}}$ neurons just before the response appears to be unaffected by motion coherence when averaged over many neurons (Figure 1F).^10^

### The heterogeneity of neuronal responses is not evident in control tasks

The memory-guided saccade task was used to identify $T_{\text{in}}$ neurons and, historically, elucidate their hallmark visual, memory, and perisaccadic responses.^37–39^ We wondered whether the heterogeneity we identified in the random dot motion task would be evident in the memory-guided saccade task. Similar persistent activity is observed across clusters (Figure S5B). We calculated the spike counts in the last 200 ms before the fixation point was extinguished and computed the average difference in standardized counts between trials with memory saccades to the left and right targets. This measure fails to differentiate the three clusters ($p_{\text{min}}>0.2$, Wilcoxon rank-sum test).

The monkeys also performed a passive motion viewing task with strong motion (c=±51.2%). Firing rates were significantly greater for leftward than rightward motion (p=0.047,0.0026, and 0.0145 for clusters 1, 2, and 3, respectively; one-sided Wilcoxon signed-rank test), but decoding accuracy was poor and similar across clusters (Figure S5C). We computed the standardized spike rate difference (excluding the initial 200 ms) between leftward and rightward motion trials, which failed to separate the clusters ($p_{\text{max}}>0.25$, Wilcoxon rank-sum test). The capacity of this measure to distinguish motion direction was low (AUC = 0.55 ± 0.01, mean ± SE across neurons). As an additional control, we repeated the decoding and clustering analyses after excluding the $T_{\text{in}}$ neurons that significantly discriminated between leftward and rightward motion in the passive motion-viewing task (N=45), yielding qualitatively similar results (Figure S6). We conclude that the response features that distinguish the three clusters of $T_{\text{in}}$ neurons are not elucidated by memory saccades or passive motion viewing. Instead, the discriminating feature is more likely associated with the decision mechanism.

### Clusters 1 and 3 are the most informative about the accuracy of the choice

**Figure 5.**
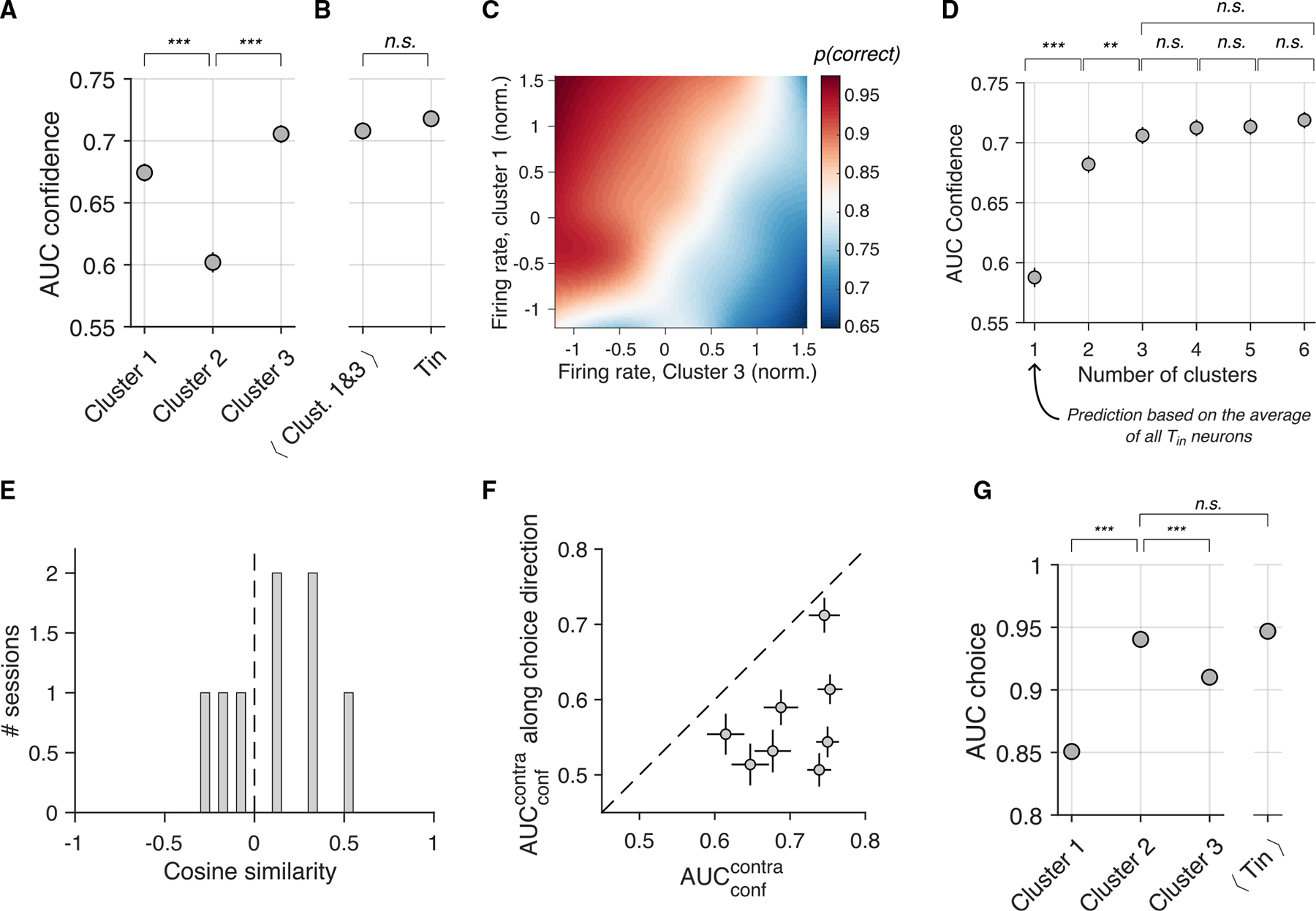
Choice accuracy information differs across clusters (A) Cluster 3 neurons are the most informative about choice accuracy. ${\text{AUC}}_{\text{conf}}$ is computed from the $T_{\text{in}}$ neurons in each cluster (abscissa). (B) ${\text{AUC}}_{\text{conf}\;}$values from accuracy decoders trained on (1) the average activity of cluster 1 and 3 neurons or (2) individual $T_{\text{in}}$ neurons. (C) Proportion of correct responses as a function of firing rates in clusters 3 (abscissa) and 1 (ordinate). Spike counts in the presaccadic window were Z scored within sessions, pooled across sessions, and grouped into 5 percentiles. Correct response rates were computed for percentile combinations and smoothed via cubic interpolation. (D) Clustering analysis applied to the regression coefficients obtained from Equation 9, using different numbers of clusters (abscissa). We averaged the spike counts in the presaccadic window of neurons belonging to the same cluster and computed ${\text{AUC}}_{\text{conf}}$ (ordinate) using Equation 6. No significant ${\text{AUC}}_{\text{conf}}$ difference was found between N=3 and N>3 clusters (bootstrap). (E) Cosine similarities between regression weights for choice ($\beta_{\text{choice}}$) and accuracy ($\beta_{\text{conf}}$) across sessions. The set of weights define coding directions in the neuronal state space. (F) Neural activity projected onto coding directions defined by $\beta_{\text{choice}}$ and $\beta_{\text{conf}}.\;{\text{AUC}}_{\text{conf}}$ was computed from these projections. Choice accuracy information is greater for projections onto $\beta_{\text{conf}}$ (abscissa) than $\beta_{\text{choice}}$ (ordinate). Each point represents a session. Error bars: SE (bootstrap). (G) ${\text{AUC}}_{\text{choice}}$ values derived from four separate regression analyses, incorporating neurons from clusters 1, 2, or 3 (left), or the average activity across all $T_{\text{in}}$ neurons (right). Error bars are ±1 SE (bootstrap). Neurons in cluster 2 are more informative about choice than those in cluster 1 or 3 and are equally informative as the average activity of all $T_{\text{in}}$ neurons. The conventions for significance levels (n.s. $p>0.05{,}^{***}p<10^{-6}$, and $**p<10^{-3}$) and error bars (SE) are the same for (A), (B), (D), and (G). Significance levels and SEs were obtained using a bootstrapping procedure.

Because confidence covaries with evidence strength and decision time, we reasoned that neurons in clusters 1 and 3 should be the most informative about choice accuracy. We tested this by repeating the logistic regression analysis (Equation 1) for each cluster separately. As expected, neurons in clusters 1 and 3 were more predictive of choice accuracy than those in cluster 2 (Figure 5A) ($p_{\text{max}}<10^{-8}$, bootstrap).

A regression model using only the mean spike counts from clusters 1 and 3 neurons achieved the same accuracy as one using all $T_{\text{in}}$ neurons (p=0.16, bootstrap; Figure 5B). Higher cluster 1 activity and lower cluster 3 activity corresponded to a greater proportion of correct choices (Figure 5C). Thus, two numbers per trial (the average activity of cluster 1 and cluster 3 neurons) capture most of the accuracy information in $T_{\text{in}}$ neurons.

We chose three clusters for analytical convenience, as regression coefficients vary continuously (Figure 4A). This number suffices, as adding more clusters did not enhance accuracy prediction, while fewer clusters reduced it (Figure 5D). Three clusters may be adequate for our purposes, as three is the minimum number required to capture the central tendency and both signs of diversity. We emphasize, however, that we do not interpret this as evidence of three distinct neuron classes within the $T_{\text{in}}$ population.

The average activity of $T_{\text{in}}$ neurons reflects motion strength during the first half of the presaccadic window (Figure 1F), which might raise concerns that the ability to distinguish correct from incorrect decisions based on the population response is primarily driven by this average activity. Three observations rule out this possibility: (1) when using a single cluster (N=1) to train the accuracy decoder, effectively relying on the within-trial average activity of all $T_{\text{in}}$ neurons, the decoding capacity is low (Figure 5D); (2) excluding cluster 2 neurons—whose average activity closely resembles that of the full population—has little impact on the ability to decode choice accuracy (Figure 5B); and (3) training the decoder using spike counts from the second half of the presaccadic window produces the same confidence signatures (Figure S2C). Therefore, the decoding capacity arises from more than just the average neuronal activity.

### Distinct contribution of $T_{\text{in}}$ neurons to decoding choice and accuracy

We assessed whether the decoding of choice accuracy and the decoding of the choice itself use the same weightings of the activity of $T_{\text{in}}$ neurons. To this end, we fit a regression model similar to the one used for accuracy prediction (Equation 1), but here, the variable to predict is choice (left/right) (Equation 7). We used spike counts in the presaccadic window to derive the best-fitting regression weights, $\beta_{\text{choice}}$. These weights define a coding direction (CD) in state space, where each $T_{\text{in}}$ neuron represents a different dimension. Unsurprisingly, choice can be decoded with high precision (${\text{AUC}}_{\text{choice}}=0.96\pm 0.014$; mean ± SE across sessions). More interestingly, the cosine similarity between the choice CD and accuracy CD is low: the average absolute value across sessions is 0.25 ± 0.05, indicating that the two directions in state space are closer to orthogonal than similar (Figure 5E). The projection of population activity onto the choice CD is barely informative about accuracy (${\text{AUC}}_{\text{conf}}=0.57\pm 0.024$; mean ± SE across sessions) and significantly less informative than the projection onto the confidence CD (p=0.0009,t test; Figure 5F).

We reasoned that the distinct contributions of $T_{\text{in}}$ neurons to decoding choice and accuracy may be evident at the cluster level. We repeated the logistic regression analysis, now training the decoder to predict choice using signals from one cluster at a time. As shown in Figure 5G, neurons from cluster 2 are more predictive of choice than those in clusters 1 and 3 ($p_{\text{max}}<10^{-8}$, bootstrap). Unlike what was observed with the accuracy decoder, the within-trial average activity of all $T_{\text{in}}$ neurons (one count per trial) is just as informative as the most predictive cluster (Figure 5G). Thus, while choice can be decoded from pooled activity, accuracy decoding relies on the heterogeneity of neuronal responses across the population.

### Multiple timescales of evidence accumulation represented by $T_{\text{in}}$ neurons

We considered whether $T_{\text{in}}$ response heterogeneity reflects differences in how persistently momentary evidence influences neuronal activity. We evaluated this by analyzing responses to short (100 ms) motion pulses in the random dot motion stimulus. For each $T_{\text{in}}$ neuron i, we counted spikes emitted from t to $\left(t+100\right)$ ms (relative to pulse onset), standardized separately by motion coherence and 100 ms window, and computed the mean difference, $\Delta {\tilde{R}}_{i}(t)$, between leftward and rightward pulses.

**Figure 6.**
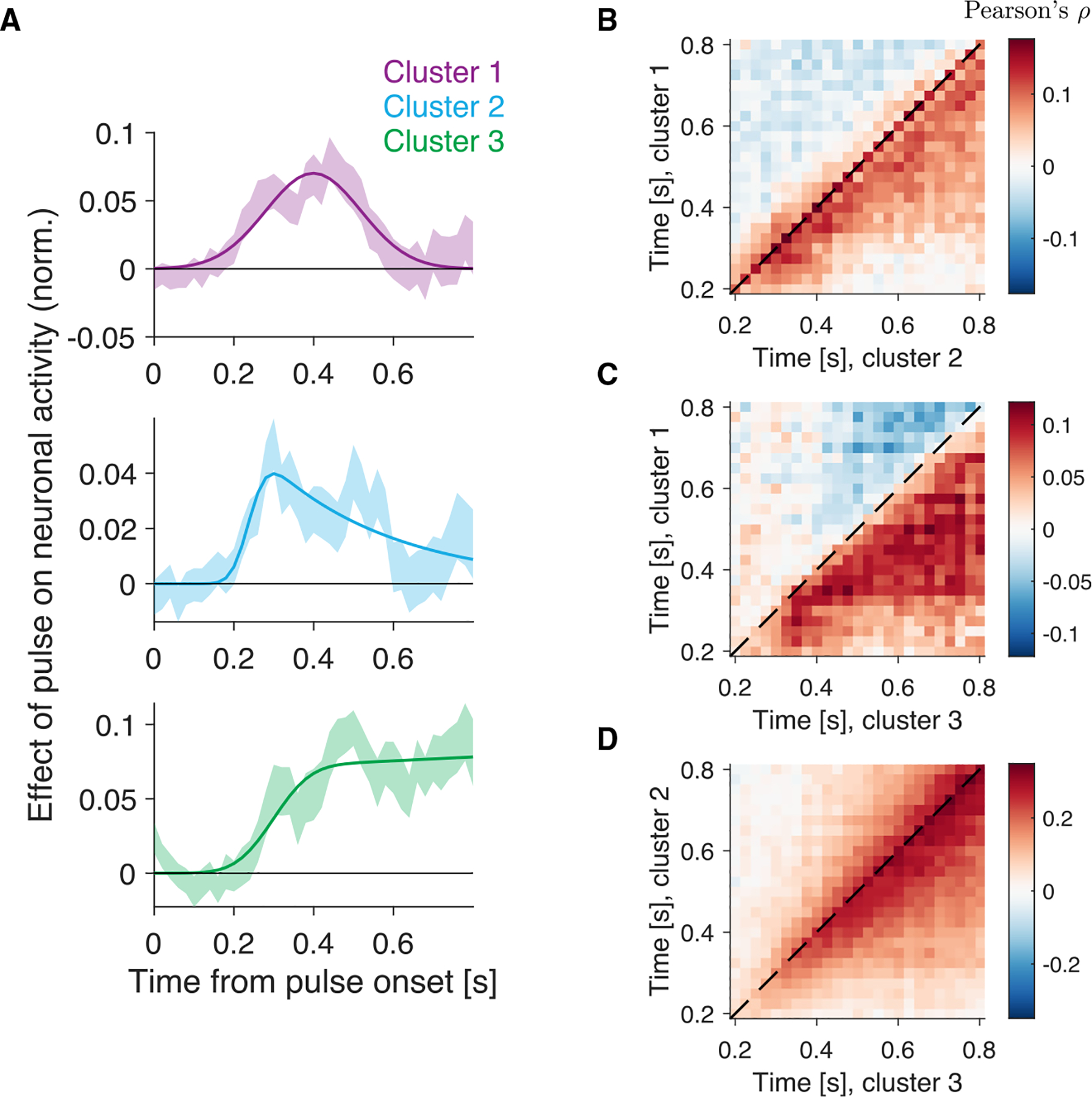
Different time constants of evidence integration by $T_{\text{in}}$ neurons (A) Influence of a brief motion pulse on the activity of cluster 1 (top), cluster 2 (middle), and cluster 3 (bottom) neurons (Equations 10 and 11). Shaded regions represent SE (bootstrap). Solid lines are function fits (see STAR Methods). Pulse effects emerge with latency ≈200 ms. (B-D) Noise correlations between neurons from clusters 1 and 2 (B),1 and 3 (C), and 2 and 3 (D). Time is relative to pulse onset. Correlation coefficients were computed in non-overlapping 25 ms windows, using trials with contralateral choices.

The effect of motion pulses on neuronal activity differs across clusters. We averaged $\Delta {\tilde{R}}_{i}(t)$ across neurons within each cluster and fit a decay function^44^ (Equation 13; Figure 6A). The bestfitting decay rates (α) were 100, 3.1, and −0.17 for clusters 1, 2, and 3, respectively, indicating increasing persistence from cluster 1 to cluster 3. Negative α (cluster 3) reflects an increase in pulse influence over time. Bootstrapping confirmed this ordering: cluster 1α exceeded cluster 2 in 86.8% of samples and cluster 3 exceeded cluster 2 in 96.6%.

We further examined correlations between clusters. Spikes were counted in 25 ms bins, forming pairs {x,y}, where $x={\tilde{S}}_{\text{Tin}}^{k}\left(t_{x}\right)$ and $y={\tilde{S}}_{\text{Tin}}^{j}\left(t_{y}\right)$, with k and j representing different clusters. The tilde in these expressions indicates the use of standardized residual values for each motion coherence. Heatmaps (Figures 6B–6D) show trial-by-trial correlations. Cluster 1 fluctuations predicted later fluctuations in clusters 2 and 3 $\left(p<10^{-8}\right)$. Similarly, cluster 2 fluctuations predicted later fluctuations in cluster 3 $\left(p<10^{-8}\right)$. Together, these findings suggest that the time constant of integration increases with ascending cluster number.

### Representation of momentary motion evidence by $T_{\text{in}}$ neurons

LIP neurons with response fields that overlap the random dot motion stimulus often display direction selectivity.^45^ The Steinemann et al.^11^ dataset also contains such neurons, termed $M_{\text{in}}$—for motion in the response field. For the $M_{\text{in}}$ neurons, the firing rate traces associated with different motion coherences are predominantly parallel (Figure S7A). They do not resemble the ramplike dynamics associated with evidence accumulation. Some $M_{\text{in}}$ neurons show selectivity for contraversive motion $\left(M_{\text{in}}^{\text{left}}\right)$ and others for ipsiversive motion ($M_{\text{in}}^{\text{right}}$) (Figures S7A–S7C, top and bottom, respectively).

In contrast to $T_{\text{in}}$ neurons, $M_{\text{in}}$ neurons do not show persistent activity in the memory-guided saccade task (Figure S7B) but do show direction selectivity in the passive motion viewing task (Figure S7C). However, during the random dot motion task, $M_{\text{in}}$ and $T_{\text{in}}^{k=1}$ neurons display similar responses in that they are direction selective but represent neither evidence accumulation nor decision termination. Note the similarity of the traces between the $M_{\text{in}}$ neurons (Figure S7A) and the $T_{\text{in}}^{k=1}$ neurons (Figure 4D, top).

We wondered if the $T_{\text{in}}^{k=1}$ neurons would share signals with the $M_{\text{in}}$ neurons despite the different locations of their response fields. We tested this idea using a noise correlation analysis similar to that shown in Figures 6B–6D but where the correlations are calculated between $T_{\text{in}}^{k=1}$ and $M_{\text{in}}$ neurons. That is, $x={\tilde{S}}_{\text{Tin}}^{k=1}\left(t_{x}\right)$ and $y={\tilde{S}}_{\text{Min}}^{\text{left}}\left(t_{y}\right)-{\tilde{S}}_{\text{Min}}^{\text{right}}\left(t_{y}\right)$. The heatmap (Figure S7D) illustrates the correlation of these residuals across trials. Correlations are stronger for off-diagonal elements where the activity of the cluster 1 neurons lags the activity of the $M_{\text{in}}$ neurons by approximately 100 ms. Correlations are significantly higher for $t_{x}>t_{y}$ than for $t_{y}>t_{x}\left(p<10^{-8}\right.$; permutation test). That is, fluctuations in the activity of $M_{\text{in}}$ neurons predict changes in $T_{\text{in}}^{k=1}$ neurons at later times. The finding is consistent with the idea that $M_{\text{in}}$ neurons drive $T_{\text{in}}^{k=1}$ neurons, although a common information source with different latencies cannot be excluded.

### Information about choice and accuracy evolves over time

Up to now, our focus has been on the epoch at the end of the decision. Here, we examine choice and accuracy signals outside the presaccadic window to characterize their time course. We construct two population signals by projecting neural activity onto the CDs defined by $\beta_{\text{choice}}$ and $\beta_{\text{conf}}$. Neural activity is obtained by binning $T_{\text{in}}$ spike counts in 100 ms sliding windows. We compute the AUC from projections onto the choice and accuracy CDs, indicating how well they discriminate between left/right choices and correct/incorrect choices, respectively.

Both AUC values peak near the time of reporting (Figure S8). Unsurprisingly, the choice predictions better distinguish left from right choices than the accuracy predictions distinguish correct from incorrect choices. We assessed whether accuracy information lags choice using a latency analysis with a bilinear “dogleg” function.^44^ Choice information diverged from baseline at 0.165 ± 0.02 s from motion onset; accuracy diverged at 0.187 ± 0.04 s (Figure S8). This difference was not significant (in bootstrapping, choice lagged confidence in 24.8% of samples). Thus, information about choice and accuracy is practically contemporaneous.

### The presaccadic confidence signal accommodates an informative prior

In the random dot motion task, confidence is influenced not only by RT and motion strength but also by the prior probability (base rate) of the different response alternatives.^46^ We ask whether the neural representation of choice accuracy that we identified is also sensitive to manipulations of prior probability. We reanalyzed data from Hanks et al.,^47^ in which the prior probability that the motion was rightward or leftward was varied in blocks of ~400 trials.

**Figure 7.**
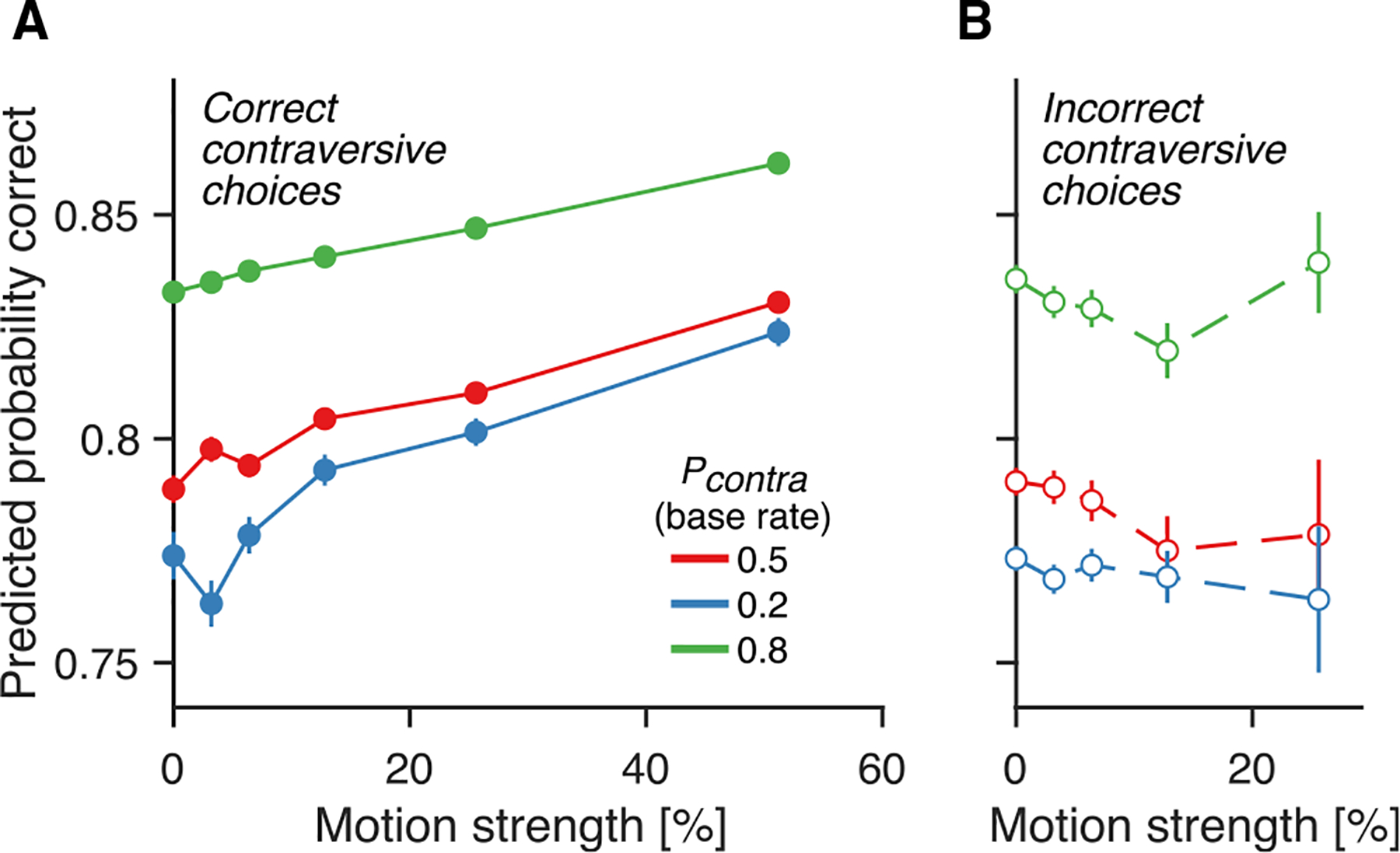
The confidence signal accommodates an informative prior (A) Probability correct inferred from individual LIP neurons from the experiment of Hanks et al.^47^ Monkeys performed blocks where the prior probability of the contralateral target being rewarded ($p_{\text{contra}}$) was 0.5, 0.2, or 0.8. Predicted probability correct increases as a function of the prior (color) and motion strength (abscissa). (B) Same as (A) but for incorrect choices. In both (A) and (B), only trials where the monkey chose the contralateral target (neurons’ response field) are included. Error bars: SEM across trials.

We decoded choice accuracy using the same approach used for the Neuropixels data. In the experiment of Hanks et al.,^47^ only one $T_{\text{in}}$ neuron was recorded per session; hence, the predictions are less informative. Nevertheless, the predicted choice accuracy is greater for trials in which the monkey chose the target with the greater base rate (Figure 7). This holds for each level of motion strength and for both correct and incorrect decisions (Figures 7A and 7B, respectively), consistent with behavioral observations.^46^ Therefore, the neural representation of choice accuracy is not only informed by motion strength and RT (Figure 3) but also by the prior probability of the chosen option (Figure 7), thus furthering the idea that the $T_{\text{in}}$ neurons support the computation of confidence.

## DISCUSSION

### Representation of confidence by $T_{\text{in}}$ neurons

Leading models of choice, RT, and confidence assume that confidence depends on the state of the losing race at the moment of choice—the balance of evidence hypothesis.^19,23,24^ This assumption stems from the idea that the evidence for the chosen alternative reaches an upper bound at decision termination and thus cannot provide a graded prediction of accuracy. However, this reasoning is incorrect. Using high-density macaque Neuropixels recordings, we confirm that while the population firing rate of $T_{\text{in}}$ neurons is stereotyped at decision termination, the heterogeneity in their firing rate dynamics is capable of supporting an estimate of confidence.

The LIP $T_{\text{in}}$ neurons exhibit persistent activity associated with saccadic intention and directed attention. Their population average firing rate represents the decision variable mediating choice and RT.^11,35^ However, not all $T_{\text{in}}$ neurons reach a stereotyped activity level at decision termination, and their deviations from the average are consistent across decisions. This heterogeneity reflects systematic differences in single-neuron dynamics.

The nature of this heterogeneity is elucidated by a clustering analysis. Cluster 1 reflects motion strength, cluster 3 shows a gradual increase in firing rate over time, and cluster 2 is largely independent of motion strength and RT at contraversive choice termination. Clusters 1 and 3 predict accuracy as effectively as the full population. Differences in response to motion pulses suggest distinct integration time constants (Figure 6), potentially supported by varying levels of recurrence in the neural circuit.^17,40,48,49^

Weight vectors derived from decoding accuracy or choice exhibit low cosine similarity. However, this does not mean distinct subsets are dedicated exclusively to choice or confidence. Instead, we favor the interpretation that choice is informed by all $T_{\text{in}}$ neurons. This is supported by (1) regression coefficients forming a continuum (Figures 4A–4D), (2) similar responses across clusters in mapping tasks (Figure S5), and (3) the trial-average activity of the $T_{\text{in}}$ neurons (a single value per trial) predicting choice as well as all neurons in cluster 2 (Figure 5G). The misalignment between choice and accuracy weights suggests that neurons encoding neither RT nor motion strength do not contribute to accuracy decoding (Figures 5A–5G). High-density recordings from the LIP and downstream areas may clarify the relationship between choice and confidence signals.

### Behavioral and neural features of confidence

The presaccadic confidence signal we identified mirrors features of human confidence reports in similar tasks, including dependencies on motion strength, prior probability, accuracy, and RT.^24,46^ Monkey confidence, assessed through betting, displays similar properties.^29^ Accounting for these features is thought to require knowledge of decision time and/or accumulated evidence for the unchosen alternative.^19,20,22,24^ We show that the necessary information is present in activity of the $T_{\text{in}}$ neurons at decision termination.

The presaccadic confidence signal may account for features of confidence not explored here, including the positive evidence bias (PEB), where confidence weights positive evidence (PE) more than negative evidence (NE).^31,50,51^ The PEB can arise if the winning race (1) assigns greater weight to PE than NE and (2) contributes more to confidence than the losing race.^31,33,34^ Our results support the latter requirement. Future work could test whether the putative confidence signal is more sensitive to PE in tasks where PE and NE are independently manipulated.^44,52^

Neurophysiological studies have identified confidence signals in the superior colliculus,^53^ pulvinar,^54^ orbitofrontal cortex,^55^ LIP,^29,56^ and other regions. Kiani and Shadlen^56^ suggested that monkey sure-bet choices rely on confidence, computed from the state of the decision variable and the duration of the stimulus. The LIP contributes by encoding the decision variable via $T_{\text{in}}$ neuron activity. We extend this by showing that the heterogeneity of $T_{\text{in}}$ neurons can support a linear readout of expected accuracy, providing a more explicit representation of confidence than previously thought.^57^

### Limitations of the study

Our study reveals that confidence in a decision can be estimated at decision termination as a weighted average of LIP neuron activity overlapping the chosen target. That said, the surprising fact that this information is present does not imply that the monkey exploits it. Testing this would require further experiments.^58^ Three factors mitigate this concern: we (1) used simple, linear decoders, which implies that it is easy for downstream areas to read confidence from the activity of $T_{\text{in}}$ neurons, (2) validated the prediction against many behavioral features of confidence, and (3) focused on a specific moment at the end of the deliberation when these neurons are broadcasting important information (e.g., where to move the eyes) to downstream areas.

An obvious limitation of the study is the absence of a behavioral assay for the monkey’s confidence, thus precluding a direct comparison between neural activity and behavioral confidence reports within individual animals. Confidence reports cannot be directly collected from non-human animals and must instead be inferred through indirect measures, such as betting behavior, willingness to wait for a reward, or opting out of a choice. An advantage of our design is that it demonstrates the presence of neural signals sufficient to estimate confidence, even in monkeys that were never explicitly trained to report it or use it for subsequent decisions.^59^ This also ensures that the observed neural representations are not confounded by the behavioral planning required to report confidence. However, our design precludes direct comparisons between neural activity and behavioral confidence reports within individual animals.

Another limitation is the inability to separate confidence in a correct decision from reward expectation, a common issue in neurophysiological confidence studies. Disentangling these factors may require tasks where reward magnitude varies independently.^60–64^ Fan et al.^61^ found that both accuracy and reward expectation are represented in the caudate and frontal eye fields. High-density population recordings in such tasks could clarify whether and how $T_{\text{in}}$ neuron heterogeneity supports accuracy estimation, reward expectation, or both.

Our study also underscores the importance of characterizing single-neuron activity even when conducting population-level analyses. The heterogeneity of neuronal responses was observed within a small population of LIP neurons ($T_{\text{in}}$) that share the property of displaying persistent activity when the monkey plans a saccade toward the contralateral choice target. This property was identified in a mapping task separate from the main task. Without this characterization, the heterogeneity we identified would not bear on the predictions of the balance of evidence hypothesis. Therefore, single-neuron and population-level analyses might best be conceived as complementary rather than competing views.^65^

## STAR★METHODS

### EXPERIMENTAL MODEL AND SUBJECT DETAILS

#### Non-human primates

This study analyzed electrophysiological and behavioral data from previously published studies involving Macaca mulatta (rhesus monkeys), conducted in compliance with institutional and national ethical guidelines.

The dataset from Steinemann, Stine et al.^11^ and Stine et al.^35^ included recordings from two male rhesus monkeys. All training, surgical, and experimental procedures followed the guidelines of the National Institutes of Health and were approved by the Institutional Animal Care and Use Committee at Columbia University. A head post and two recording chambers were implanted using aseptic surgical techniques under general anesthesia. The LIP chamber placement was guided by structural MRI.

The dataset from Hanks et al.^47^ included two rhesus monkeys (gender unspecified). All training, surgical, and experimental procedures adhered to the National Institutes of Health Guide for the Care and Use of Laboratory Animals and were approved by the University of Washington Animal Care Committee.

#### Human participants

This study reanalyzed previously published human participant data from van Den Berg et al.^22^ The original study was conducted in accordance with ethical guidelines established by the Cambridge Psychology Research Ethics Committee, which approved the experimental protocol. The dataset included four human participants (age range: 21–34 years; gender unspecified) who performed a random dot motion (RDM) discrimination task, reporting both choice and confidence simultaneously. All participants provided informed consent before participation. No sample size calculation was performed. Allocation of subjects to experimental groups: not applicable.

### METHOD DETAILS

#### Behavioral tasks

##### Random dot motion task

In the main task, the monkeys had to decide the net direction (leftward or rightward) of a circular patch of limited-lifetime, dynamic random dots and report their choice when ready by making a saccadic eye movement from the central fixation to the left or right choice target. The monkey initiates at trial by directing the gaze to a central fixation point. After 0.25–0.7 s (truncated exponential with time constant λ=0.15s), two red choice targets (diameter 1 dva; degrees of visual angle) are presented in the left and right visual fields. After a random delay (0.25-0.7s,λ=0.4s), the random dot motion stimulus is displayed until the monkey initiates a saccadic eye movement to report its choice.

The random dot motion comprises limited lifetime dots displayed within a circular area (diameter 5 dva) centered on the fixation point. The dot density is 16.7 dots · dva^−2^ s^−1^. The direction and strength of the motion are chosen pseudorandomly on each trial, such that the coherence, C∈{±0%,±3.2%,±6.4%,±12.6%,±25.6%,±51.2%}. The sign of C determines the direction of motion; positive values indicate leftward motion. For C=0%, the sign indicates the random direction that is rewarded on that trial. The absolute value |C| establishes the motion strength: the probability that a dot displayed in video frame n is displaced by Δx in frame n+3 (i.e., 40 ms later). Otherwise the dot is replaced by a new dot at a random position. The displacement, Δx=±0.2dva, is consistent with apparent motion speed of 5 dva per s (see^10^ for further details). Monkeys are rewarded for making a saccadic eye movement to the correct choice target. On trials with 0% motion coherence, either saccadic choice is rewarded with a probability of 0.5. Incorrect responses are penalized by increasing the inter-trial interval by up to 3 s (see^35^ for further details). On approximately half of the trials, the motion coherence is incremented or decremented for 100 ms by 4% coherence for monkey M and 3.2% for monkey J.^35^ The onset time of the pulse is chosen randomly from a truncated exponential distribution: $t_{\text{min}}=0.1\;s$ to $t_{\text{max}}=0.8\;s$ from motion onset (λ=0.4s). Monkey M completed 9,684 trials across five sessions, while Monkey J completed 8,142 trials in three sessions.

##### Control tasks

Monkeys also completed two additional tasks in each session: a passive motion viewing task and a memory-guided saccade task. In the passive motion viewing task, the monkey views ± 51.2% coherent motion for 0.5 s (and for 1 s on a small number of trials in one session). The task matches the main task but without choice targets. The monkey is rewarded for maintaining fixation during the motion presentation.

In the memory-guided saccade task,^38,66^ a target was briefly flashed (200 ms) at a pseudo-random location in the visual field. After a variable delay (0.2–0.9 s for monkey M,λ=0.3s;0.3-1.3s for monkey J,λ=0.2s), the fixation point was extinguished and the monkey had to make a saccadic eye movement to the remembered location of the target. The monkey was rewarded if the gaze was within ± 2.5 degrees of visual angle of the target location.

##### Biased prior probability task

The analysis of prior probabilities makes use of previously published single neuron recordings from two other monkeys that performed the same task as Steinemann, Stine et al.^11^ However, Hanks et al.^47^ varied the prior probability that the rewarded choice was left or right. In blocks of trials, the sign of the motion coherence was biased in favor of positive or negative. They also included blocks with a neutral (i.e., uninformative) prior. In blocks with neutral priors, both targets had an equal 50% chance of being correct. In biased conditions, one direction had an 80% probability of being correct and the other had a 20% probability of being correct, except for a small number of sessions in one monkey where a 67–33% prior was used (these data were not included in our study nor in Hanks et al.^47^).

Sessions typically began with a block of 200–400 trials under a neutral prior. The monkeys then completed 300–600 trials in which the prior favored one of the targets, with the most-likely target being the one chosen least often during the neutral prior block. To signal to the monkeys which target was more likely, each biased block was preceded by 20 trials of 100% coherent motion toward the more likely target. These trials were not included in our analysis. In some sessions, monkeys completed an additional block with a prior favoring the opposite target. See Hanks et al.^47^ for details.

##### Combined choice-confidence task in humans

van Den Berg et al.^22^ asked participants to discriminate the direction of motion of a random dot motion display similar to that of Steinemann, Stine et al.^11^ and Hanks et al.^47^ Subjects held the handle of a vBOT manipulandum used to record the position of the handle at 1,000 Hz.^67^ A horizontal mirror projecting a downward facing CRT monitor prevented subjects from seeing their arm. A chin rest ensured that the viewing distance was approximately 40 cm.

Participants reported choice and confidence simultaneously by moving the handle to one of four circular targets displayed at the corners of a 17 cm × 17 cm square. The two targets on the left corresponded to a leftward motion choice, and the two on the right corresponded to a rightward motion choice. In half of the blocks, the two top targets corresponded to a high-confidence choice and the bottom targets to a low-confidence choice; in the other half, the mapping was reversed such that the bottom targets corresponded to high-confidence and the top targets to high-confidence. To motivate participants to make calibrated confidence reports, the high- and low-confidence targets had different payoffs for correct and incorrect choices. The low confidence targets gave a 1 point reward for a correct choice and a 1 point loss for an incorrect choice. The high-confidence target gave 2 points for a correct choice and a loss of 3 points for an incorrect choice.

Figure 3A reproduces data from a representative participant (Subject 2 in Figure 2 of van Den Berg et al;^22^), who completed 9,023 trials over 12 experimental sessions. Data from the other participants is shown in Figure S1. Details of the experimental procedure should be sought in the original publication.^22^

#### Neurophysiological recordings (LIP)

##### Main task

Steinemann, Stine et al.^11^ used a prototype “alpha” version of Neuropixels1.0-NHP45 probes (developed by IMEC and HHMI-Janelia) to record multiple single-unit activities in the ventral part of area LIP (LIPv). Steinemann, Stine et al.^11^ used anatomical MRI to localize LIPv and used single-neuron recordings (Thomas Recording GmbH) to verify that the activity conformed to known physiological properties of LIPv before proceeding with multi-neuron recordings. The Neuropixels probes are equipped to record from 384 of the 4,416 available electrical contacts distributed along their 45 mm long shaft. Data was only recorded from the 384 contacts closest to the probe tip (Bank 0), covering 3.84 mm. The reference and ground signals were directly connected to each other and to the monkey’s headpost. A total of 1,084 neurons were recorded over eight sessions, with each session yielding between 54 and 203 neurons (see Table 2 of Steinemann, Stine et al;^11^ for details).

##### Biased prior probability task

Hanks et al.^47^ recorded fifty-two neurons from the LIP area of two rhesus monkeys. Recordings were made using standard methods for extracellular recording of action potentials from single neurons.^10^ See Hanks et al.^47^ for details.

### QUANTIFICATION AND STATISTICAL ANALYSIS

#### Race model of decision making

In the race model, two drift-diffusion processes, $x_{L}(t)$ and $x_{R}(t)$, compete until one of them reaches an upper bound. The first to reach the upper bound determines choice and RT. $x_{R}$ accumulates evidence for right minus left, and $x_{L}$ accumulates evidence for left minus right. The dynamics of the decision variables is described by the following difference equations:

(Equation 2) $$x_{L}^{\left(t+1\right)}=x_{L}^{\left(t\right)}+\kappa \Delta t\left(C+C_{0}\right)+u^{\left(t+1\right)}+\eta_{L}^{\left(t+1\right)}\sqrt{\Delta t},$$

(Equation 3) $$x_{R}^{\left(t+1\right)}=x_{R}^{\left(t\right)}-\kappa \Delta t\left(C+C_{0}\right)+u^{\left(t+1\right)}+\eta_{R}^{\left(t+1\right)}\sqrt{\Delta t}$$

where κ is a measure of the signal-to-noise, Δt=0.005s is the time step, C is the motion coherence (positive for leftward motion), C_0_ is a bias term and η is zero-mean normally distributed noise with unit variance. $\eta_{L}(t)$ and $\eta_{R}(t)$ are sampled from a bivariate Normal distribution such that the correlation between them is ρ=-0.7. At time $t=0,\;x_{L}=x_{R}=0$.

The urgency signal $u^{\left(t+1\right)}$ decreases the amount of evidence needed to trigger a response as time progresses.^47^ For times t<d, the value of $u^{\left(t+1\right)}$ is zero, indicating no urgency. For times greater than $d,\;u^{\left(t+1\right)}$ assumes a constant value equal to aΔt, where a is a parameter that represents the linear rate of rise of the urgency signal.

The decision variables in the model cannot drop below a lower reflective bound. If an update to a decision variable would result in a value lower than $B_{\text{reflect}}$, the variable’s value is set to $B_{\text{reflect}}$ for that time step. That is:


(Equation 4) $$x_{L}^{\left(t+1\right)}\leftarrow \text{max}\left(x_{L}^{\left(t+1\right)},B_{\text{reflect}}\right),$$

(Equation 5) $$x_{R}^{\left(t+1\right)}\leftarrow \text{max}\left(x_{R}^{\left(t+1\right)},B_{\text{reflect}}\right).$$

The lower, non-absorbing bound simply instantiates the fact that firing rates must be ≥ 0.

The decision terminates when one of the races reach an upper bound at B. The choice is leftward (rightward) if $x_{L}\left(x_{R}\right)$ reaches the bound first. The decision time is the time taken to reach the bound. The RT is the sum of the decision time and a non-decision time, which is normally distributed with a mean of $\mu_{nd}$ and standard deviation $\sigma_{nd}$.

The free parameters of the model are $\Theta =\left\{\kappa ,B,a,d,C_{0},\mu_{nd},\sigma_{nd}\right\}$. We use simulations to fit them to data. Data from each monkey were fit separately. For a given set of parameters, we simulated 10 times as many trials as were completed by the monkey. For each combination of choice and motion coherence, we fit the distribution of decision times obtained from the model with an Epanechnikov (parabolic) kernel to obtain a smooth probability density function of decision times. The distribution of decision times is convolved with the distribution of non-decision times to obtain a probability density function of RTs. We compute a separate p.d.f. of RTs for each combination of choice and motion coherence, and use them to calculate the likelihood of the parameters given the single-trial choice and RT data. We use BADS^68^ to find the maximum-likelihood parameters. The best-fitting parameters are shown in Table S1.

#### Data analysis

##### Preprocessing of neuronal data

Our study focuses on $T_{\text{in}}$ neurons, i.e., those that show persistent activity during saccade planning to the target contralateral to the recording site. For the Neuropixels data, $T_{\text{in}}$ neurons were identified post hoc using a memory-guided saccade task.^11,35^ Hanks et al.^47^ used the same task to identify neurons with spatially selective persistent activity and to place targets within the response field of these neurons.

Unless otherwise stated, neurophysiological analyses are based on the number of spikes emitted by each $T_{\text{in}}$ neuron in the 100 ms epoch that ends 50 ms before the initiation of the saccadic eye movement used to report the choice. We refer to this time interval as the presaccadic window.

##### Accuracy decoder

In each of the 8 sessions, we trained two decoders to predict the accuracy of the monkey’s choice, using only contralateral (left) or ipsilateral (right) choices, respectively. We trained the decoder using simple logistic regression:

(Equation 6) logitpcorrect=βconfS⊤x+β0,

where $S_{x}={\tilde{S}}_{\text{Tin}}$ is the standardized spike count of each $T_{\text{in}}$ neuron in the interval from 150 ms to 50 ms before saccade onset. Standardization (i.e., Z score) is applied to each neuron independently. The fitted coefficients, $\beta_{\text{conf}}$, establish a vector the same size as the number of simultaneously recorded $T_{\text{in}}$ neurons. We refer to this vector as a coding direction in neuronal state space. $\beta_{0}$ is a constant that captures the accuracy rate over all stimulus conditions, which is typically much better than chance (typically, $p_{\text{correct}}>0.7$).

We apply the same strategy to decode from subpopulations of neurons by redefining $S_{x}$. For example, Figure 5A shows the result of applying the regression model (Equation 6) to the subset of $T_{\text{in}}$ neurons belonging to each cluster. For the analysis of the data from Hanks et al.,^47^ $S_{x}$ contains the standardized spike counts of the single $T_{\text{in}}$ neurons recorded in separate experiments. We use the same presaccadic window as in the analysis of the Neuropixels data.

To derive the confidence estimates, the decoders are trained using 10-fold cross-validation. The data are divided into 10 groups, each containing an approximately equal number of trials selected randomly. One group is used as the prediction set, while the remaining nine groups are used for training. This process is repeated 10 times, ensuring that the confidence estimates for each trial are based on a prediction. For the analyses shown in Figure S8, we derive $\beta_{\text{choice}}$ and $\beta_{\text{conf}}$ using all trials instead of using cross-validation. This approach allows us to obtain a single set of regression weights per session, rather than 10 sets as produced by the cross-validation method.

The confidence estimates are used to calculate the area under the ROC curve (AUC). Figure 2A exemplifies the distributions that are used for the ROC analysis. For the analyses shown in Figures 2B and 5E, AUC values were calculated separately for each session. The statistical comparisons use a one-tailed paired t test applied to the logit-transformed AUC values from each session. Elsewhere, AUCs are not computed per session, but rather the confidence estimates from all sessions are pooled before calculating a single AUC. Standard errors were computed using bootstrapping (N=5,000 samples).

We also use bootstrap samples to determine if there is a significant difference between two AUC values. For instance, to determine if the ${\text{AUC}}_{\text{conf}}$ obtained using only the cluster 1 neurons is larger than that obtained using only the cluster 2 neurons (Figure 5A), we bootstrap to obtain $N=5,\;000\;{\text{AUC}}_{\text{conf}}$ values for each cluster. We then compare the AUC values for all $N=25\times 10^{6}$ pairwise comparisons and determine significance as the proportion of comparisons for which the AUC value from cluster 1 neurons is larger than that from cluster 2 neurons.

##### Choice decoder

We use the same spike-count standardization (z-scoring), analysis time window, and cross-validation method to predict the monkey’s choice on each trial:

(Equation 7) logitpleft=βchoiceS˜⊤Tin+β0.

Here, $\beta_{0}$ reflects the monkeys bias for or against a left choice, not the monkey’s accuracy. Unlike the accuracy decoder, the choice decoder is trained on trials with both contralateral and ipsilateral choices.

##### Reaction time decoder

We also use a logistic decoder to assess whether $T_{\text{in}}$ neurons contain, just before the response, information about RT. The model is:

(Equation 8) logitpfast=βRTS˜⊤Tin+β0.

It was fit independently for each session using only trials with contraversive (left) choices. Fast and slow responses were defined relative to the median RT. We use the same cross-validation method that we used for the accuracy and choice decoders.

##### Latency analysis

We used the cumulative sum (CUSUM) method to determine the latency of direction-selective responses in $T_{\text{in}}$ neurons.^43^ Receiver operating characteristic (ROC) analysis was used to assess the directional selectivity of each $T_{\text{in}}$ neuron. The area under the curve (AUC) in this analysis represents the degree separation—from 0.5 (complete overlap) to 1 (complete separation)—between the spike count distributions for leftward and rightward motion in single trials. The AUC was calculated from spike counts in the interval 100–400 ms after motion onset. We restricted the analysis to correct trials with RTs greater than 450 ms and motion coherence greater than 10%. For neurons with an AUC greater than 0.55, we calculated the difference in spike counts (in 25 ms bins) between leftward and rightward choice trials. These differences were then added cumulatively over time, as required by the CUSUM method. Typically, the cumulative difference remains around zero prior to the onset of direction selectivity, and then gradually increases or decreases depending on the neuron’s preferred choice.

To determine the onset of direction selectivity, we fit a “dogleg” function to the cumulative spike sum. This function starts with a flat line from $t_{0}=0$ and transitions to a linear increase or decrease starting at $t_{1}>t_{0}$. The end of the flat portion of the fit, which occurs between 0 and 500 ms after the onset of motion, was considered the latency. Using cumulative sums of spikes to estimate latencies helps reduce the effect of neural noise. The fitting step further reduces the influence of the number of trials on latency estimates, providing an advantage over traditional methods such as t-tests in moving windows.^44^ The significance of the difference in latency between neurons from different clusters was assessed with a two-tailed *t test*.

We conducted a similar analysis to estimate the latencies depicted in Figure S8. We construct bootstrap samples combining the time course of AUC values from individual sessions (N=5,000 bootstrap samples). A dogleg function was fit to each bootstrap sample, resulting in 5,000 latency estimates. The arrows in Figure S8 identify the mean latency across the bootstrap samples. The p-value we report is the proportion of bootstrap samples for which the choice signal deviates from baseline later than the confidence signal.

##### Clustering

We use linear regression to explain the spike counts of each $T_{\text{in}}$ neuron in the presaccadic window. As independent variables, we used motion coherence (C), choice, RT, and an offset:

(Equation 9) $${\tilde{S}}_{\text{Tin}}^{\left(i\right)}=\beta_{0}^{\left(i\right)}+\beta_{1}^{\left(i\right)}C+\beta_{2}^{\left(i\right)}\text{RT}+\beta_{3}^{\left(i\right)}\text{choice}.$$

${\tilde{S}}_{\text{Tin}}^{\left(i\right)}$ is the standardized spike count of neuron i in the aforementioned time interval. The regression analysis was performed separately for each $T_{\text{in}}$ neuron, including correct trials only.

We then apply k-means clustering, using 3 clusters, to the regression coefficients associated with motion coherence, RT and choice. The cluster labels (1–3) were chosen so that the average of $\beta_{1}$ is smallest for cluster 1 neurons, intermediate for cluster 2 and largest for cluster 3. We confirmed the robustness of the neuron cluster assignments by independently deriving them using either the odd or even trials (Figure S3). We also fit a regression model similar to Equation 9, but without the choice and RT terms and considering only correct contralateral choices. The regression coefficients associated with motion coherence are shown in Figure 4D.

##### Motion pulses

We estimate the time course of the effect of the brief motion pulses on neuronal activity by aligning the spike times of each $T_{\text{in}}$ neuron to the onset of the motion pulse. For each time -60<t<800ms relative to the onset of the motion pulse, we calculate the number of spikes emitted in the time epoch between t and (t+100)ms. Time t is advanced in steps of $20\;\text{ms}.R_{i}(t,j)$ contains the spike counts for neuron i emitted at time t from pulse onset, in trial j. Only trials where the RT is at least 150 ms greater than t, and those with a motion pulse ($\approx \frac{1}{2}$) are included in the analysis. We then standardize the spike counts independently for each motion coherence, to obtain ${\tilde{R}}_{i}(t,j)$. We average ${\tilde{R}}_{i}(t,j)$ across trials with a leftward (contraversive) pulse, and trials with a rightward (ipsiversive) pulse, and calculate the difference, left minus right, between these averages, to obtain $\Delta {\tilde{R}}_{i}(t)$.

$\Delta {\tilde{R}}_{i}(t)$ approximates for each $T_{\text{in}}$ neuron i and time t, the influence of the motion pulse on neuronal activity. We average $\Delta {\tilde{R}}_{i}(t)$ across the $N_{K}$ neurons belonging to the same cluster K,

(Equation 10) $$\Delta {\tilde{R}}_{K}\left(t\right)=\frac{1}{N_{K}}\underset{i\in K}{\sum }\Delta {\tilde{R}}_{i}\left(t\right),$$

and normalize the average subtracting a baseline,

(Equation 11) $$D_{K}(t)=\Delta {\tilde{R}}_{K}(t)-\text{baseline},$$

where baseline is the average of $\Delta {\tilde{R}}_{K}(t)$ for times t between 0 and 0.2 s. Figure 6A shows $D_{K}(t)$ for the three clusters.

We use a curve-fitting approach to estimate the rate at which the effect of the motion pulse dissipates over time. We fit $D_{K}(t)$ using a function f(x) constructed on the following two assumptions: (*i*) the onset latency of the effect of the motion pulse on neuronal activity follows a Normal distribution, (*ii*) the effect dissipates exponentially. Given these assumption, the differential equation describing the time course of f(t) is

(Equation 12) $$\frac{\partial f(t)}{\partial t}=-\alpha f(t)+𝒩(t|\mu ,\sigma ),$$

where μ and σ are the mean and standard deviation of the Normal distribution (𝒩), and α is the reciprocal of the time constant of the dissipation. The solution to this equation is:

(Equation 13) $$f\left(t\right)=d\cdot \text{exp}\left(\mu \alpha +\frac{1}{2}\sigma^{2}\alpha^{2}-\alpha t\right)\cdot \Phi \left(t|m,\sigma \right),$$

where d is a scaling parameter, and Φ(⋅∣m,σ) is the cumulative Gaussian distribution with mean $m=\mu +\sigma^{2}\alpha$ and standard deviation σ. The fit function has four parameters: {d,μ,σ,α}. We fit the parameters to minimize the sum over time points of the squared differences between f(t) and $D_{K}(t)$.

We compared the best-fitting dissipation parameter (α) across clusters. We generated bootstrap samples (N=5,000) by selecting with replacement from the pool of neurons that belong to a given cluster. For each of these samples, we compute the parameter α. We evaluate the significance of the difference in α values in the data by the proportion of bootstrap samples that result in a difference in α values as extreme as the one we observed in the data.

##### Cross-correlation analysis

The analysis depicted in Figures 6B–6D is based on the spike counts of neurons from clusters 1–3. Spike counts were calculated in 25 ms windows, aligned to motion onset, up to 50 ms before the RT. We compute standardized residuals separately for each time bin, motion coherence and session. Standardized residuals were combined across sessions. The processed signals are referred to as ${\tilde{S}}_{\text{Tin}}^{k=1},\;{\tilde{S}}_{\text{Tin}}^{k=2}$ and ${\tilde{S}}_{\text{Tin}}^{k=3}$. We then calculated the Pearson correlation coefficient between every pair of signals (Figures 6B–6D), for every pair of time steps between 0.2 and 0.8s.

We used permutation tests to asses statistical significance. We define two regions of interest based on the time from stimulus onset in the x and y dimensions (Figure 6B). ROI1 is defined by $t_{x}>t_{y}$, and ROI2 is defined by $t_{y}>t_{x}$, for time time points shown in Figure 6B. If y causally affects x, or if y and x receive a common input but the integration time constant is greater for y than for x, then the pairwise correlations between x and y should be greater in ROI1 than in ROI2. We calculated the difference in correlations between two groups, $\left\langle {\rho \;}_{\text{ROI1}}\right\rangle -\left\langle \rho_{\;\text{ROI2}}\right\rangle$, where the expectation is calculated over the time bins within each region of interest (ROI). This difference was then contrasted with those obtained after randomly shuffling the order of trials for one of the dimensions ($N_{\text{shuffles}}=200$). Significance was determined by the probability of achieving a difference as extreme as the one observed in the experimental data.

The same procedure was applied to the cross-correlation analysis shown in Figure S7D, but with $x={\tilde{S}}_{\text{Tin}}^{k=1}\left(t_{x}\right)$ and $y={\tilde{S}}_{\text{Min}}^{\text{left}}\left(t_{y}\right)-{\tilde{S}}_{\text{Min}}^{\text{right}}\left(t_{y}\right)$, where ${\tilde{S}}_{\text{Min}}^{\text{left}}(t)$ and ${\tilde{S}}_{\text{Min}}^{\text{right}}(t)$ are the standardized residuals obtained from the activity of the $M_{\text{in}}$ neurons preferring contraversive and ipsiversive motion, respectively.

## Supplementary Material

1

## Figures and Tables

**Table:** KEY RESOURCES TABLE

REAGENT or RESOURCE	SOURCE	IDENTIFIER
Deposited data		
Neural & behavioral	Steinemann et al.^11^	https://doi.org/10.5281/zenodo.7946011
Software and algorithms		
Original code	This paper	https://doi.org/10.5281/zenodo.14908233
MATLAB	MathWorks	https://www.mathworks.com/products/matlab.html

## Data Availability

Further information and requests for resources should be directed to and will be fulfilled by the lead contact, Ariel Zylberberg (ariel.zylberberg@gmail.com). This study did not generate new unique reagents. This study did not generate new data. Original code has been deposited at GitHub (https://github.com/arielzylberberg/LIP_Confidence_CellReports2025) and is publicly available as of the date of publication. Any additional information required to reanalyze the data reported in this paper is available from the lead contact upon request.

## Highlights

- Mean firing rates of a population of LIP neurons reach a common level at the end of a decision
- Choice accuracy can be predicted from the heterogeneous dynamics of these neurons
- Accuracy decoded from neural activity mirrors confidence patterns in humans
- Choice, reaction time, and confidence may be represented by the same population of neurons
