# Supplementary material for: A population representation of the confidence in a decision in the parietal cortex
Source: Cell Rep. Author manuscript; Available in PMC 2025 May 17. (PMC12084876; doi:10.1016/j.celrep.2025.115526)
Supplement: 1 [file NIHMS2076727-supplement-1.pdf]

Cell Reports, Volume *44*

Supplemental information

A population representation of the confidence in a decision in the parietal cortex

Ariel Zylberberg and Michael N. Shadlen

Supplemental information

"A Population Representation of the Confidence in a Decision in the Parietal Cortex"

Ariel Zylberberg and Michael N. Shadlen

	κ	B	$a[s^{-1}]$	$d[s]$	ρ	$\mu_{nd}[s]$	$\sigma_{nd}[s]$	C_0	B_{rectif}
Monkey M	14.86	1.73	1.63	0.13	-0.7	0.28	0.07	0.01	-1
Monkey J	12.97	0.88	0.57	0.62	-0.7	0.3	0.03	0	-1

Table S1. Best-fitting parameters of the race model. ρ and B_{rectif} were not fit but fixed to predefined values.

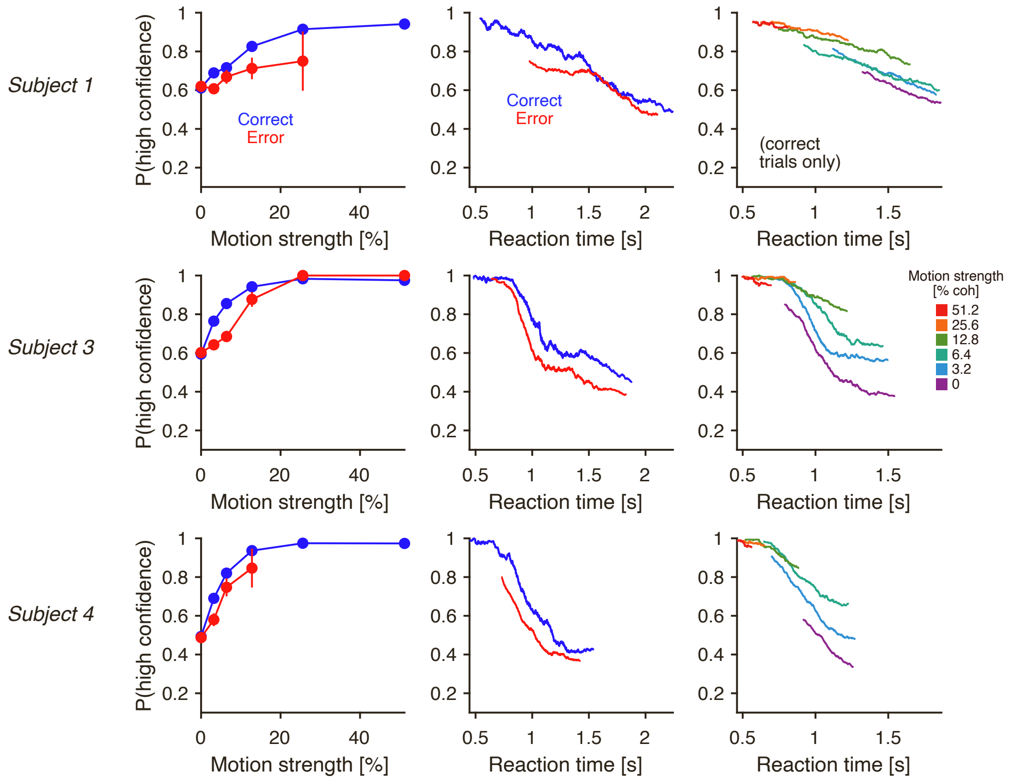

Figure S1. Signatures of confidence in the data of van Den Berg et al. [10]
Same as Fig. 3A for the other three participants in van Den Berg et al. [10].

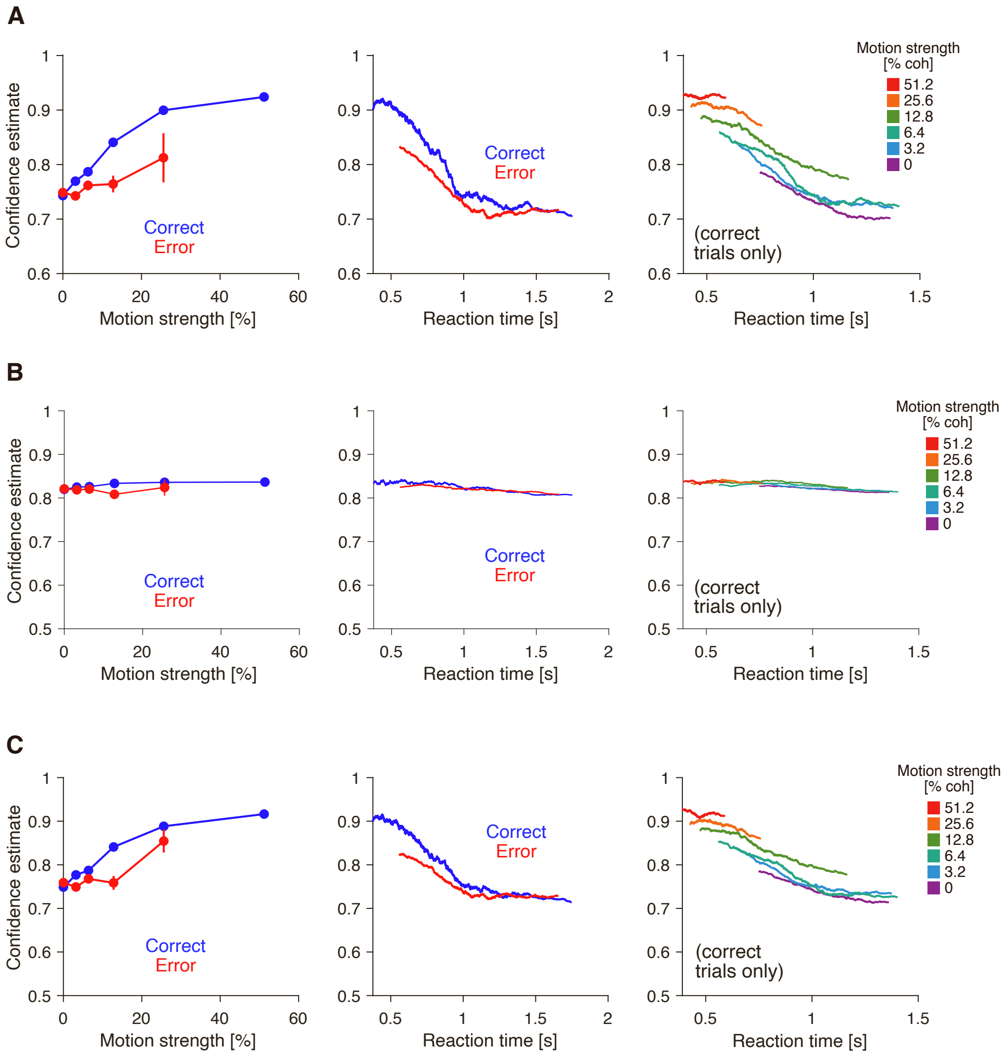

Figure S2. Choice accuracy inferred from neural activity
(A) Same as Fig. 3B, except that the confidence estimates obtained from the accuracy decoder are not thresholded into high and low confidence categories. Results are qualitatively similar to those obtained from the behavioral data (Fig. 3A). **(B)** Same as panel A, but for the decoding analysis using gaze data before and after the saccadic eye movement as predictors, instead of neural signals. **(C)** Same as panel A, but using a narrower presaccadic window (from 100 ms to 50 ms before the choice report).

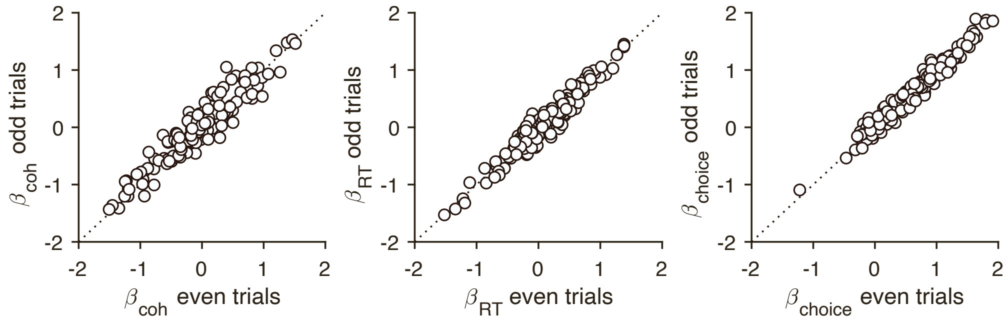

Figure S3. Consistency of the neural representation of motion coherence, RT and choice
We use only the odd or even trials to explain the standardized (z-scored) spike counts of each neuron in the presaccadic window as a function of motion coherence, RT, and choice. The figure shows the best-fitting regression coefficients plotted against each other. Each data point corresponds to a different T_{in} neuron. Panels 1-3 correspond to the best-fitting regression coefficients for motion coherence, RT, and choice, respectively. The regression coefficients are highly consistent across independent regression analyses.

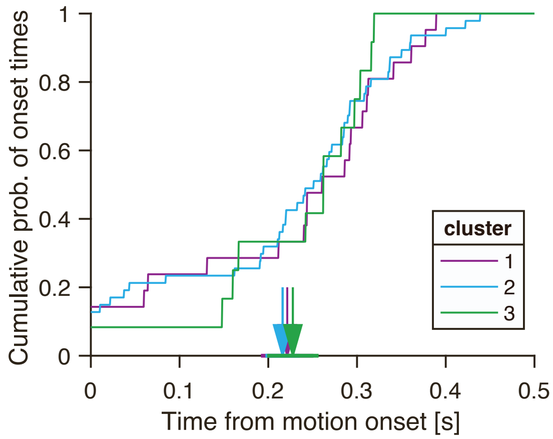

Figure S4. Latency to motion selectivity of individual T_{in} neurons
For each T_{in} neurons separately, we calculate the latency to motion direction selectivity using the CUSUM method[13]. The cumulative distribution of onset times is shown separately for neurons belonging to the three clusters. The vertical arrows indicate the mean onset time for each cluster, and the horizontal line indicates the standard error of the mean.

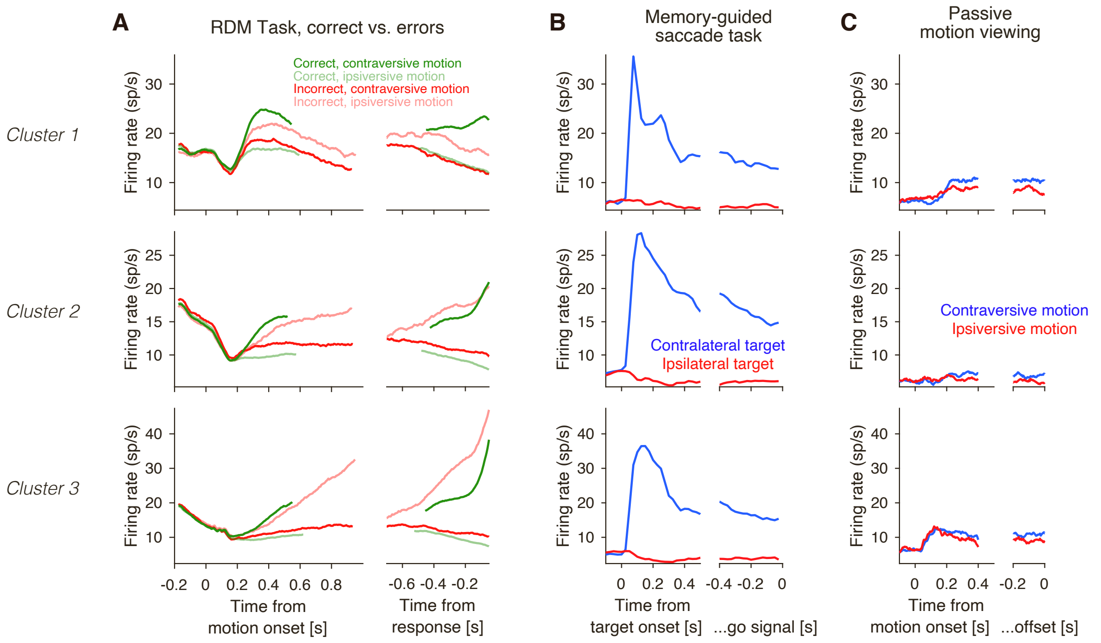

Figure S5. Mean response of neurons from each cluster for correct and incorrect decisions, memory-guided saccades, and passive motion-viewing tasks

(A) Random dot motion task. Neurons from all three clusters exhibit stronger responses to contralateral (leftward) choices compared to ipsilateral choices, regardless of whether the choice is correct (green) or incorrect (red). **(B)** Memory-guided saccade task. Blue and red traces correspond to saccades to the target located contralaterally and ipsilaterally, respectively. The horizontal gray bar indicates the time of target presentation. In the panels on the right, neural activity is aligned to the go signal (i.e., the offset of the fixation point). The average time from target onset to the go signal is 0.82 seconds. **(C)** Passive motion–viewing task. Blue and red traces correspond to contraverise (leftward) and ipsiversive (rightward) motion, respectively. Traces are aligned to the onset (left) and offset (right) of the random dot motion stimulus.

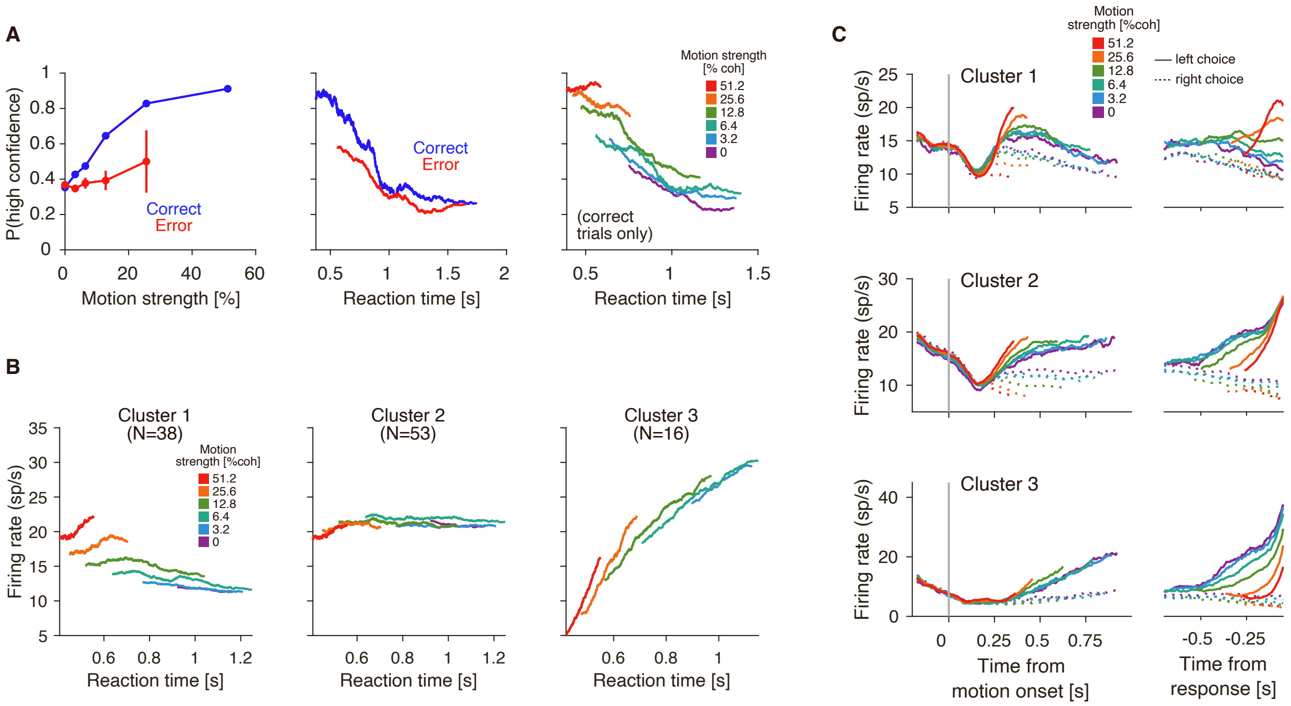

Figure S6. Decoding and clustering without the T_{in} neurons with significant modulation in the passive motion-viewing task. We repeated the decoding and clustering analyses without the T_{in} neurons that significantly discriminated between leftward and rightward motion in the passive motion viewing task. Significance was assessed using a Wilcoxon rank–sum test comparing spike rates on leftward and rightward motion trials. Spike rates were calculated for each trial in the epoch between 0.2s after motion onset and motion offset. Neurons with p-values lower than 0.05 were deemed significant. The results of the decoding and clustering analyses are qualitatively similar to those obtained without excluding these neurons. **(A)** Analysis equivalent to that shown in Fig. 3B. **(B)** Analysis equivalent to that shown in Fig. 4B. **(C)** Analysis equivalent to that shown in Fig. 4C.

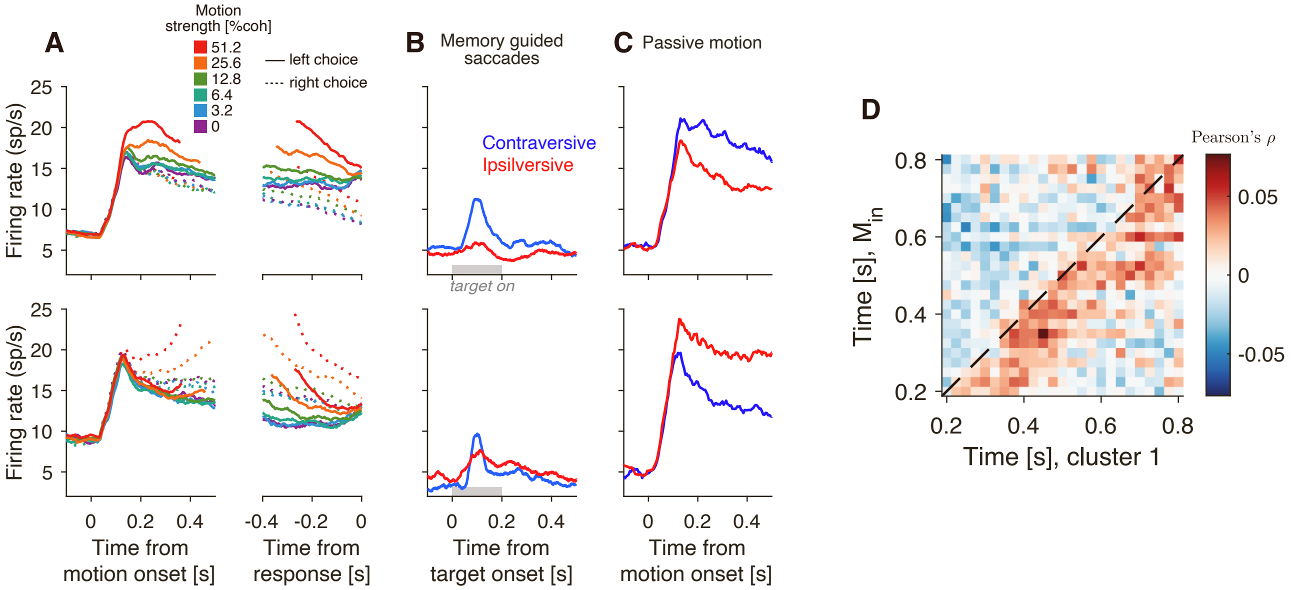

Figure S7. Momentary motion evidence in LIP
(A–C) Firing rate of the M_{in} neurons in the random dot motion task (**A**), the memory-guided saccade task (**B**), the passive motion viewing task (**C**). The upper (lower) row represents M_{in} neurons that prefer leftward (rightward, respectively) motion. **(D)** Noise correlations between the motion-selective neurons with the motion stimulus on their response field (ordinate), and the $T_{in}^{k=1}$ neurons (abscissa). Same conventions as in Fig. 6B–D.

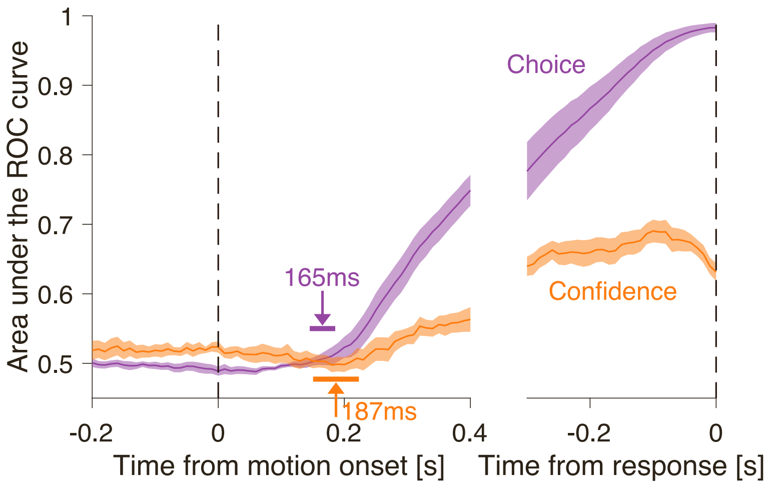

Figure S8. Contemporaneous decoding of choice and accuracy
Time-course of the AUC values obtained from the projection of the neuronal activity along the directions defined by β_{choice} (purple) and β_{conf} (orange). Shading indicates s.e. across sessions. Projections were calculated in 100 ms windows in steps of 10 ms. The arrows indicate the time when the traces first deviate from baseline (see Methods), and the associated horizontal bars are the s.e. of these estimates.
